# Stochasticity, Nonlinear Value Functions, and Update Rules in Learning Aesthetic Biases

**DOI:** 10.3389/fnhum.2021.639081

**Published:** 2021-05-10

**Authors:** Norberto M. Grzywacz

**Affiliations:** ^1^Department of Psychology, Loyola University Chicago, Chicago, IL, United States; ^2^Department of Molecular Pharmacology and Neuroscience, Loyola University Chicago, Chicago, IL, United States

**Keywords:** reinforcement learning, aesthetic value, value function, delta rule, regret minimization, stochastic dynamics

## Abstract

A theoretical framework for the reinforcement learning of aesthetic biases was recently proposed based on brain circuitries revealed by neuroimaging. A model grounded on that framework accounted for interesting features of human aesthetic biases. These features included individuality, cultural predispositions, stochastic dynamics of learning and aesthetic biases, and the peak-shift effect. However, despite the success in explaining these features, a potential weakness was the linearity of the value function used to predict reward. This linearity meant that the learning process employed a value function that assumed a linear relationship between reward and sensory stimuli. Linearity is common in reinforcement learning in neuroscience. However, linearity can be problematic because neural mechanisms and the dependence of reward on sensory stimuli were typically nonlinear. Here, we analyze the learning performance with models including optimal nonlinear value functions. We also compare updating the free parameters of the value functions with the delta rule, which neuroscience models use frequently, vs. updating with a new Phi rule that considers the structure of the nonlinearities. Our computer simulations showed that optimal nonlinear value functions resulted in improvements of learning errors when the reward models were nonlinear. Similarly, the new Phi rule led to improvements in these errors. These improvements were accompanied by the straightening of the trajectories of the vector of free parameters in its phase space. This straightening meant that the process became more efficient in learning the prediction of reward. Surprisingly, however, this improved efficiency had a complex relationship with the rate of learning. Finally, the stochasticity arising from the probabilistic sampling of sensory stimuli, rewards, and motivations helped the learning process narrow the range of free parameters to nearly optimal outcomes. Therefore, we suggest that value functions and update rules optimized for social and ecological constraints are ideal for learning aesthetic biases.

## Introduction

Values and in particular aesthetic ones are a significant part of our lives because they contribute to our process of decision making (Skov, 2010). Because humans are highly social animals, the set of values of each person must be in tune with their cultures and surroundings. Therefore, learning is an essential component of how our values come to be. In the case of aesthetic values, they begin to be learned early on in life, such that by preschool age, cultural idiosyncrasies are observed in children (Senzaki et al., 2014). In addition, these values continue to progress over our lifespans (Park and Huang, 2010).

How does the brain learn aesthetic values? An important meta-analysis of neuroimaging considered commonalities of aesthetic biases across multiple sensory modalities (Brown et al., 2011). The results of this and many other imaging studies indicated general mechanisms for appraisal involving a well-studied (Schultz, 1998, 2016) reward-based learning circuit (Lacey et al., 2011; Vartanian and Skov, 2014; Wang et al., 2015). However, these studies suggest that many independent factors impact this process of reward-based learning, with Brown et al. (2011) in particular discussing a novel role for motivation.

Because the development of aesthetic biases involves a rewards-based learning circuitry, a mechanism akin to reinforcement learning (O’Doherty et al., 2003; Sutton and Barto, 2018) likely mediates the process. Several theoretical frameworks for aesthetic values have elements of reward-circuitry and reinforcement learning. Some of these theories are computational (Martindale, 1984; Schmidhuber, 2010; Van de Cruys and Wagemans, 2011; Aleem et al., 2019, 2020) and some are not (Biederman and Vessel, 2006; Skov, 2010; Vessel and Rubin, 2010; Chatterjee and Vartanian, 2014). Of the computational theories, the only one considering motivation is that of Aleem et al. This is also the only theory studying the temporal evolution of learning. Simulations of a model based on the Aleem et al. theoretical framework and mathematical analysis lead to three main findings. First, different people may develop distinct weighing of aesthetic variables because of individual variability in motivation (Nelson and Morrison, 2005; Brown and Dissanayake, 2009; Silvia et al., 2009). Demonstration of the development of individuality is especially important in a theory in which learning leads to a degree of coordination of aesthetic values across society. Second, individuals from different cultures and environments may develop different aesthetic values because of unique sensory inputs and social rewards. Third, because learning is stochastic stemming from probabilistic sensory inputs, motivations, and rewards, aesthetic values vary in time.

A potential problem for reinforcement-learning models for the brain is the linearity of many of the most important mechanisms. For example, the model used by Aleem et al. (2020) assumes a linear value function (Sutton and Barto, 2018), that is, a linear relationship between sensory inputs and values. Furthermore, this model makes a linearity assumption for the update rule of the value function. Thus, although the reward has a nonlinear dependence on sensory inputs, brain actions would approximate this dependence linearly. Biologically, these linear mechanisms are not reflective of typical reward-related neural signaling (Schultz, 2015). Moreover, recent studies have signaled the need for a new conception of aesthetics that incorporates distributed processing and nonlinear recurrent networks (Leder and Nadal, 2014; Nadal and Chatterjee, 2019). Assuming such linear mechanisms is common even in Machine Learning to lighten computations and mathematical analysis (Chung et al., 2018). In addition, linear methods have also been well-explored theoretically (Tsitsiklis and Van Roy, 1997; Maei, 2011; Mahmood and Sutton, 2015; Iigaya et al., 2020) and empirically (Dann et al., 2014; White and White, 2016) in the Machine Learning literature. Finally, arguments have been made that linear rules perform comparably to deep neural networks when predicting subjective aesthetic values (Iigaya et al., 2020). However, modeling nonlinear processes with linear approximations should produce errors, or equivalently, regret in Machine Learning terminology (Kaelbling et al., 1996; Sutton and Barto, 2018; formally, regret is the difference between an agent’s performance with that of an agent that acts optimally). Hence, increasing effort has begun in Machine Learning to develop methods for nonlinear value functions (Tesauro, 2005; Xu et al., 2007; Kober et al., 2013; Gu et al., 2016; Osband et al., 2016; Chung et al., 2018).

In this article, we present mathematical and computational analyses of linear and nonlinear reinforcement-learning models for the acquisition of aesthetic values. We analyze 16 models. They stem from the combination of four types of value function (one linear and three nonlinear) and four types of value-function update rule (two making a linearity assumption for the updates and two assuming nonlinearities). All these models incorporate motivation (Brown et al., 2011). The comparisons between the models use different metrics, the most important of which is “regret.” We measure regret as the difference between reward and the prediction of reward. We choose this metric because humans often experience emotional responses to regret as a decision error (Gilbert et al., 2004; Filiz-Ozbay and Ozbay, 2007; Somasundaram and Diecidue, 2016). Another metric is time of convergence, which is important because a good learning mechanism should acquire its values as quickly as possible.

## Theoretical Considerations

We have split the description of the theoretical considerations into two subsections, general and mathematical. The “General Description of the Theoretical Considerations” section has a description of the ideas without any equations. Our goal in that section is to help the reader understand the elements of the theoretical considerations at an intuitive level. That section may allow some readers to skip the equations (“Mathematical Description of the Theoretical Considerations” section) and the “Materials and Methods” section, and go directly to the “Results” section. The subsections of “General Description of the Theoretical Considerations” and “Mathematical Description of the Theoretical Considerations” sections have parallel titling. The parallel subjects of these subsections may help the reader when connecting the intuitive and mathematical levels of understanding.

### General Description of the Theoretical Considerations

#### Motivation-Gated Reinforcement Learning of Aesthetic Values

The starting point for the analyses in this article is the theoretical framework of Aleem et al. (2020). The core of the framework is reinforcement learning. As it is normal for reinforcement learning, the system first receives inputs from the external world, that is, the sensory inputs. Moreover, the system receives internal inputs on the motivation to act. The system then uses these external and internal inputs to estimate what will be the expected reward during the period in which these signals are arriving. This estimate is commonly referred to as value. When rewards arrive, they are compared with the values (i.e., the estimated rewards). If there is a mismatch (i.e., non-zero regret), the system learns by updating the parameters of the internal model (the value function). This update allows the system to achieve its goal of producing better reward predictions in the future.

While reinforcement learning is at the heart of the theoretical framework, it has four notable extensions. First, the estimate of reward itself is equivalent to aesthetic value. Second, the reinforcement-learning circuitry includes the concept of motivation within, which, by our definition, refers to the internal drive of an individual to act given an input. More specifically, motivation is a component of the likelihood of a person to act, which in turn is akin to policy in Machine Learning (Sutton and Barto, 2018). Third, both motivation and sensory inputs to the theoretical framework are probabilistic. Fourth, the inputs to our theoretical framework depend not only on individuals but also across societies.

#### Linear and Nonlinear Value Functions

In this article, we investigate the performance of aesthetic learning with four types of value function. First, we probe the linear value function, which yields an estimate of reward that is proportional to the sensory inputs. The constants of proportionality, which Aleem et al. (2020) call aesthetic weights, are the free parameters that the process of learning should estimate. Second, we follow the linear step with a saturation function characteristic of many neurobiological processes (Hudspeth et al., 2013; Schultz, 2015). Such saturation function added to the output of the linear function models a value-function nonlinearity resulting from diminishing marginal utility (Kreps, 1990). We call this mechanism the Output-saturation model because we apply the saturating process at the output of the linear stage. Third, we apply the same saturation mechanism to each component of the linear model. Appropriately, we call this mechanism the Component-saturation model. Fourth, we use the value function developed by Aleem et al. (2020) in their theoretical framework for aesthetic learning.

#### Update Rules for Value Functions

In the Aleem et al. article, the updates of the value function are performed with the delta rule (Sutton and Barto, 2018). This rule implements a gradient descent on the magnitude of regrets (errors) of the predictions of reward. The delta rule stipulates that the change of the free parameters of the value function should be proportionate to the difference between observed and predicted rewards, typically denoted δ. Thus, the larger this difference is, the faster this change becomes. In all the simulations and mathematical analyses in this article, this component of the delta rule applies. Furthermore, the delta rule prescribes in what direction the vector of free parameters of the value function should change (Here, we often use “free parameters” when referring to the vector of free parameters of the value function). This change should be in the direction opposite to the gradient of the value function with respect to this vector. If the value function is linear, then this gradient is equal to the vector of sensory stimuli (Sutton and Barto, 2018).

However, the standard delta rule has some disadvantages, suggesting an important modification. To understand these disadvantages, let us start with some of the advantages of this rule. The first worth mentioning is that it attempts to minimize regret. This minimization holds for both standard reinforcement learning (Sutton and Barto, 2018) and the version here with motivation gating (Aleem et al., 2020). In addition, for the linear value function, the delta rule tends to optimize the trajectory of the free parameters (Aleem et al., 2020). However, as we will illustrate in the “Hypotheses Tested in This Article” sections, this advantage does not apply in general to nonlinear value functions. Fortunately, a related rule that has this advantage does exist. This new rule points the trajectory of the free parameters directly to the closest point in the isoline corresponding to the reward received (the target isoline). Because this rule takes the vector through the shortest route, we say that the rule implements the Shortest-path strategy. We sometimes also call this the Phi rule because the vertical line in Φ bisects its ellipse with the shortest path.

#### Hypotheses Tested in This Article

**Figure 1:**
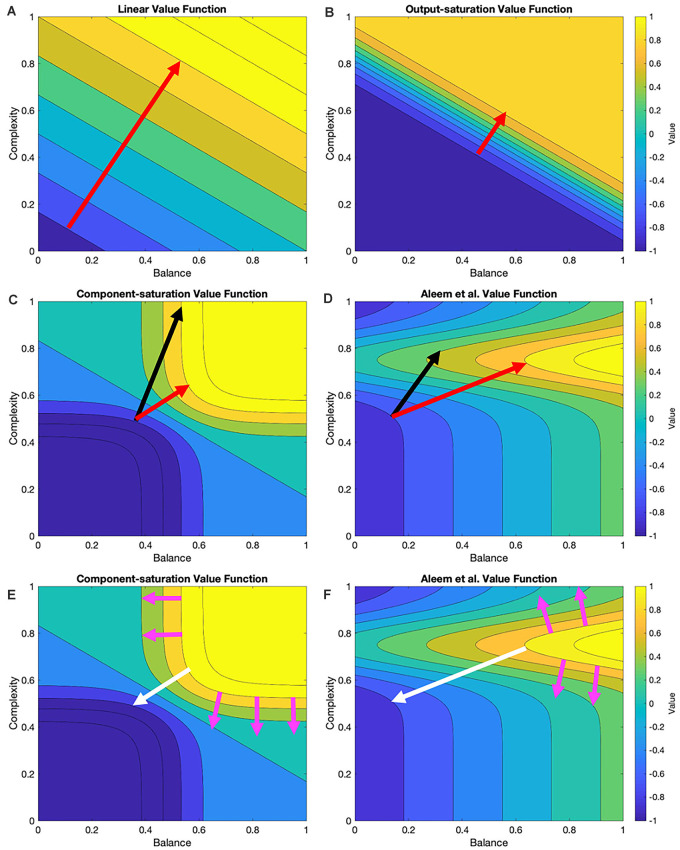
Contour plots of four examples of value functions and relationships to possible update rules. These value functions are two-dimensional in this research (“Stochastic Sampling” section) but multi-dimensional in general. In the examples given here, the two dimensions are measures of balance and complexity in visual images. These measures range from 0 to 1 for the value functions illustrated in this research. The parameters of the value functions are those of Table 2. The red arrows indicate the optimal trajectory from the current position of the vector of free parameters of the value function to the closest point of the isoline corresponding to the sampled reward (Here, we call this curve the target isoline, but in general, it is an isosurface.) The black arrows indicate the trajectory based on gradient computation. **(A,B)** For the Linear and Output-saturation value functions, the gradient and optimal trajectories coincide. **(C,D)** For the Component-saturation and Aleem et al. models, the gradient trajectory is not optimal. **(E,F)** However, if one computed the gradients from the target isoline instead of the current position (magenta and white arrows), the gradient at the optimal point on the target isoline would be parallel to the optimal trajectory (“Results” section; white arrows). We call this computation the Shortest-path or Phi rule (“Update Rules for Value Functions” section).

In this article, we probe the performance of learning under various value functions (“Linear and Nonlinear Value Functions” section) and their various update rules (“Update Rules for Value Functions” section). At the simplest level, the expectations for these probes are straightforward. For example, an update rule appropriate for a linear value function should do poorly with a nonlinear one. However, we wish to develop expectations that are more granular for the various value functions and update rules. Figure 1 helps us formulate hypotheses based on these rules and functions.

From Figure 1, if one disregards the stochastic nature of the learning process, we can draw the following seven hypotheses about the interactions between values functions and their update rules:

I. Assume that the value function is linear and the update delta rule follows the gradient at the position of the vector of free parameters. The final regret should be zero and the update convergence should be fast. After convergence, the recovery from fluctuation errors should also be fast.
II. A similar hypothesis applies to the Output-saturation value function because of its straight and parallel contour lines.
III. We hypothesize that the regret magnitude should be larger with the Component-saturation and Aleem et al. value functions than with the Linear and Output-saturation ones. The convergence and the recovery from fluctuation errors should also be slower for the former two. These problems will be especially acute for the Aleem et al. value function.
IV. Similarly, we hypothesize a straight trajectory for the Linear and Output-saturation value functions (except for small stochastic fluctuations). But the trajectory should be curved for the other two functions.
V. Instead, the Phi Rule should yield no regret and fast convergence and recovery from fluctuation errors for all value functions.
VI. Regardless of the rule, although value will reach a unique fixed point, the free parameters will not. The reason for the lack of uniqueness is that many parameter combinations yield the same value (isolines in Figure 1).
VII. Let a parameter of the model of reward have higher sensitivity coefficient than another parameter. Thus, if we increase the former parameter, we get more reward than if we raise the latter by a similar amount (Vidal et al., 1966; Saltelli et al., 2008). The corresponding free parameters of the value function should exhibit the same hierarchy of contributions to the estimation of reward.

However, if one does not disregard the stochastic nature of the learning process, these hypotheses could be wrong. With the stochastic sampling, the contour plots in Figure 1 would change across samples, possibly making the convergence more complex. The computer simulations in the “Results” section test this possibility.

### Mathematical Description of the Theoretical Considerations

#### Motivation-Gated Reinforcement Learning of Aesthetic Values

Much of the work described in this section appears in Aleem et al. (2020). We will only sketch the relevant work in that work here, leaving details to that article but pointing out the new ideas in this article.

Let the sensory inputs be a *N* dimensional vector, $\vec{u}(t),$ with the various components *u_i_* corresponding to variables that the brain uses to represent the external world. Without loss of generality, Aleem et al. assumed that 0 ≤ *u_i_* ≤ 1. Moreover, and more importantly, Aleem et al. assumed that the value function was linear. Instead, we assume a general value function

(1) $$v(t)=m(t)\mu \;(\vec{u}(t):\vec{w}(t)),$$

where 0 ≤ *m* ≤ 1 is the motivation function, *μ* is a general nonlinear differentiable function representing the fully motivated value, and $\vec{w}(t)$ is the vector of free parameters of the value function (In this research, we use the colon to indicate parameters and thus, $\mu \left(\vec{u}(t):\vec{w}(t)\right)$ means that the function *μ* has $\vec{\mu }$ as variables and $\vec{w}$ as parameters. The reason $\vec{w}$ varies with time is that learning operates by parametric optimization.) Thus, if we interpret *m* as the probability of acting around time, then the expected received reward is

(2) $$r(t)=m(t)r^{*}(t),$$

where r* is the reward that a fully motivated person would get.

Aleem et al. (2020) used a delta-rule update of the value function by first computing

(3) δ(t)=r(t)−v(t),

They then used the gradient update rule assuming a linear value function. We instead must use the value function in Equation (1), which yields

(4) $$\frac{d\vec{w}(t)}{dt}=\varepsilon \delta (t)\;\nabla_{w}\mu \;(\vec{u}(t):\vec{w}(t)),$$

where *ɛ* > 0 is a constant.

To complete the theoretical framework, we need to specify the statistical properties of $\vec{\mu }$, *m*, and r*. Following Aleem et al., we define the probability density functions

(5) $$P({\vec{I}}_{u}|\vec{B}),\;P((\vec{u}(t),r^{*}(t))|{\vec{I}}_{u}),$$

where $\vec{B}$ indicates the vector of parameters characteristic of the social and environmental background under consideration and ${\vec{I}}_{u}$ is the vector of parameters of an individual in this society. We also define the probability density function of *m* as

(6) $$P({\vec{I}}_{m}|\vec{B}),\;P(m(t)|\vec{u}(t),{\vec{I}}_{m}),$$

where we insert $\vec{B}$ to indicate that individual motivation may depend on environmental and social backgrounds.

#### Linear and Nonlinear Value Functions

For reinforcement learning to work well, the value function should be able to capture the structure of the incoming rewards. From Equations (1–3), (5) and (6), the expected least-square error (dropping both the dependence on *t* and the parameters for the sake of conciseness) is

(7) $$E={∭}_{\vec{u},r^{*},m}P(\vec{u},r^{*})P(m|\vec{u})\;{\left(m\;(\mu \;(\vec{u})-r^{*})\right)}^{2}.$$

This error is a function of the value function $\mu (\vec{u})$ (Riesz and Szökefalvi-Nagy, 1990). As shown in Appendix: Optimal Value Function, the minimal of this function occurs when

(8) $$\mu_{opt}(\vec{u}:\vec{w},\vec{k})=\langle r^{*}\rangle \left(\vec{u}:{\vec{I}}_{u}=\left[\vec{w},\vec{k}\right]\right),$$

where $\langle r^{*}\rangle \left(\vec{u}:{\vec{I}}_{u}=\left[\vec{w},\vec{k}\right]\right)$ indicates the mean of r* given the sampled sensory inputs, and the free $\left(\vec{w}\right)$ and constant $\left(\vec{k}\right)$ parameters of the value function.

We are now ready to specify the optimal value functions obtained after setting the mean rewards in our models.

##### Linear Value Function

$$\langle r_{lin}^{*}\rangle \left(\vec{u}:{\vec{I}}_{u}={\vec{w}}^{\left(lin\right)}\right)={\vec{w}}^{\left(lin\right)}\cdot \vec{u},$$

(9) $$\mu_{lin}(\vec{u}:\vec{w})=\vec{w}\cdot \vec{u},$$

where ${\overset{\leftarrow }{w}}^{\left(\mathrm{li}n\right)}$ are constant parameters of the model of reward.

##### Output-Saturation Value Function

$$\langle r_{out}^{*}\rangle \left(\vec{u}:{\vec{I}}_{u}=\left[{\vec{w}}^{\left(out\right)},\vec{k}=[\alpha_{1},\beta_{1}]\right]\right)=\frac{e^{\alpha_{1}({\vec{w}}^{\left(out\right)}\cdot \vec{u}-\beta_{1})}-1}{e^{\alpha_{1}({\vec{w}}^{\left(out\right)}\cdot \vec{u}-\beta_{1})}+1},$$

(10) $$\mu_{out}(\vec{u}:\vec{w},\alpha_{1},\beta_{1})=\frac{e^{\alpha_{1}(\vec{w}\cdot \vec{u}-\beta_{1})}-1}{e^{\alpha_{1}(\vec{w}\cdot \vec{u}-\beta_{1})}+1},$$

where ${\vec{w}}^{\left(out\right)},\alpha_{1}>0,$ and *β*_1_ are constant parameters of the model of reward. The right-hand side of Equation (10) is the hyperbolic tangent, a sigmoidal function centered on *β*_1_ and with speed of rise controlled by *α*_1_.

##### Component-Saturation Value Function

$$\langle r_{com}^{*}\rangle \left(\vec{u}:{\vec{I}}_{u}=\left[{\vec{w}}^{\left(com\right)},\vec{k}=[\alpha_{2,1},\beta_{2,1},\alpha_{2,2},\beta_{2,2}]\right]\right)=\sum_{i=1}^{N}\frac{e^{\alpha_{2,i}(w_{i}^{\left(com\right)}\cdot {\vec{u}}_{i}-\beta_{2,i})}-1}{e^{\alpha_{2,i}(w_{i}^{\left(com\right)}\cdot {\vec{u}}_{i}-\beta_{2,i})}+1},$$

(11) $$\mu_{com}\left(\vec{u}:\vec{w},\alpha_{2,1},\beta_{2,1},\alpha_{2,2},\beta_{2,2}\right)=\sum_{i=1}^{N}\frac{e^{\alpha_{2,i}\left(w_{i}\cdot u_{i}\;-\;\beta_{2,i}\right)}-1}{e^{\alpha_{2,i}\left(w_{i}\cdot u_{i}\;-\;\beta_{2,i}\right)}+1},$$

where again, ${\vec{w}}^{\left(com\right)},\alpha_{21}>0,\beta_{21},\alpha_{22}>0,$ and *β*_22_ are constant parameters of the model of reward. In turn, ${\vec{w}}^{\left(com\right)}$, *w_i_*, and *u_i_* are the ith components of the vectors ${\vec{w}}^{\left(com\right)}$, $\vec{w}$, and $\vec{u}$ respectively.

##### Aleem et al. Value Function

$$\left(\vec{u}:{\vec{I}}_{u}=\left[{\vec{w}}^{\left(ale\right)},\vec{k}=[\alpha_{3},\beta_{3}]\right]\right)=-w_{1}^{\left(ale\right)}+2w_{1}{}^{\left(ale\right)}\mu_{1}-w_{2}{}^{\left(ale\right)}\theta (\alpha_{3},\beta_{3})+w_{2}{}^{\left(ale\right)}e^{-\frac{{\left(u_{2}\;-\;\alpha_{3}\right)}^{2}}{2\beta_{3}^{3}}},$$

(12) $$\mu_{ale}(\vec{u}:\vec{w},\alpha_{3},\beta_{3})=-w_{1}+2w_{1}u_{1}-w_{2}\theta (\alpha_{3},\beta_{3})+w_{2}e^{-\frac{{\left(u_{2}-\alpha_{3}\right)}^{2}}{2\beta_{3}^{2}}}.$$

where again, ${\vec{w}}^{\left(ale\right)},\alpha_{3}>0,$ and *β*_3_ are constant parameters of the model of reward. In turn, $w_{i}^{\left(ale\right)}$ is the ith components of the vector ${\vec{w}}^{\left(ale\right)}$. Finally, the function *θ* is (*α*_3_, *β*_3_) is

$$\theta (\alpha_{3},\beta_{3})=\int_{0}^{1}e^{-\frac{{\left(u_{2}\;-\;\alpha_{3}\right)}^{2}}{2\beta_{3}^{2}}}du_{2}.$$

The derivation of Equation (12) follows from the equations in Aleem et al. (2020).

#### Update Rules for Value Functions

We use two update rules for the free parameters, with the first being the gradient-based delta rule in Equation (4). To implement this rule, we must first sample $\vec{u}(t),r^{*}(t),$ and *m* (*t*) from Equations (5) and (6) (details in “Materials and Methods” section). From, these samples, we can compute the value functions as in the second part of Equations (9)–(12) and thus, *δ* (*t*). Finally, we must compute the gradient, $\nabla_{w}\mu \left(\vec{u}(t):\vec{w}(t)\right),$ for these value functions.

The second update rule that we use in this article is what we call the Phi (or Shortest-path) rule (Figures 1E,F). To define this rule, we begin by considering

(13) $$\left\{{\vec{w}}_{r}(t)|\mu \left(\vec{u}(t):{\vec{w}}_{r}(t)\right)=r^{*}(t)\right\},$$

which is the set of all free parameters of the value function that yield the sampled reward. Thus, ${\vec{w}}_{r}(t)$ are the points of the target isolines in Figure 1. Now, define the optimal point in the target isoline, that is, the point closest to $\vec{w}$:

(14) $${\vec{w}}_{opt}(t)=\arg\min_{{\vec{w}}_{r}(t)}||{\vec{w}}_{r}(t)-\vec{w}(t)||.$$

This point may not be unique, but the lack of uniqueness is rare (and one can break it with tiny random perturbations), and thus, we neglect it here. We now define the vector $\vec{\Phi }(t)$ as

(15) $$\vec{\Phi }\left(\vec{w}(t):\vec{u}(t),r^{*}(t)\right)=\frac{{\vec{w}}_{opt}(t)-\vec{w}(t)}{\left\|{\vec{w}}_{opt}(t)-\vec{w}(t)\right\|},$$

that is, the unit vector pointing from $\vec{w}(t)$ to ${\vec{w}}_{opt}(t)$. With $\vec{\Phi }(t)$ in hand, we propose a new rule instead that in Equation (4):

(16) $$\frac{d\vec{w}(t)}{dt}=\varepsilon \delta (t)\vec{\Phi }\left(\vec{w}(t):\vec{u}(t),r^{*}(t)\right).$$

#### Hypotheses Tested in This Article

As mentioned in the “Update Rules for Value Functions” section, the gradient-based delta rule attempts to minimize regret. This minimization holds for both standard reinforcement learning (Sutton and Barto, 2018) and the version here with motivation gating (Aleem et al., 2020). In the latter study, the demonstration of the minimization of regret was for the linear value function (Equation 9). In Appendix: Minimization of Regret Under Optimal Value Functions and the Delta Rule, we extend the demonstration for nonlinear value functions in the presence of motivation. Specifically, we show that the rule in Equation (4) tends to perform a stochastic gradient descent on the error

(17) $$E={\langle m(t){\left(r^{*}(t)-\mu \left(\vec{u}(t):\vec{w}(t)\right)\right)}^{2}\rangle }_{t},$$

where ${\langle \rangle }_{t}$ stands for time average. Consequently, the rule in Equation (4) performs a gradient descend on the error of value weighed statistically by the motivation.

Another implication of the delta rule is that it tends to maximize the rate of convergence for the linear value function (Aleem et al., 2020). The delta rule also maximizes the rate of recovery from fluctuation errors after convergence. These maximizations are contingent on the gradient being perpendicular to the isolines. However as seen in Figures 1C,D, the gradient is not generally perpendicular to the isolines for nonlinear value functions.

These conclusions on the gradient-based delta rule underlie Hypotheses I–IV.

In contrast, the Shortest-path Phi rule overcomes the deficiencies of the gradient-based delta rule. The Phi rule does so by going directly to the optimal point, ${\vec{w}}_{opt},$ on the target isoline (Equations 13 and 16). Appendix: Perpendicularity Condition Under the Phi Rule adds to this conclusion, demonstrating an important property of ${\vec{w}}_{opt}$:

(18) $${\vec{w}}_{opt}-\vec{w}\propto \nabla_{w}\mu (\vec{u}:{\vec{w}}_{opt}).$$

Consequently, the perpendicular of the target isoline through ${\vec{w}}_{opt}$ is parallel to the vector connecting $\vec{w}$ to ${\vec{w}}_{opt}$. This result extends the conclusion for the delta rule that it tends to maximize the rate of convergence of $\vec{w}$ for the linear value function. The result also extends the conclusion that the delta rule tends to maximize the rate of recovery from fluctuation errors after convergence. These conclusions are now valid for nonlinear value functions if one uses the Phi rule.

## Materials and Methods

We tested the hypotheses of “Hypotheses Tested in This Article” sections through mathematical analyses and computer simulations. The “Simulated Conditions” section lists all the conditions (mixtures of value functions and update rules) simulated in this article. Then the “Methods for Computer Simulations” and “Stochastic Sampling” sections describe the technical details of the simulations. These sections are followed by a summary of the simulation procedures (“Summary of the Simulation Procedures” section) and the parameters of the simulations (“Standard Simulation Parameters” section). Finally, the “Statistics to Test the Hypotheses” section describes the statistics used to test the hypotheses. The detailed mathematical analyses are left to the appendices, but the results are explained at appropriate places in this article.

### Simulated Conditions

This article compares the performance of various value functions and their update rules in the learning of aesthetic biases. Hence, we performed simulations combining conditions of value functions and update rules. The simulated conditions appear in Table 1.

**Table 1:** Conditions simulated.

Set	Condition	Reward	Value function	Update rule
Purely linear	1	$\langle r_{\mathrm{lin}}^{*}\rangle$; Eq. (9)	*μ_lin_*; Eq. (9)	▽*_w_μ_lin_*; Eqs. (4) and (9)
	2	$\langle r_{\mathrm{out}}^{*}\rangle$; Eq. (10)	*μ_lin_*; Eq. (9)	▽*_w_μ_lin_*; Eqs. (4) and (9)
	3	$\langle r_{\mathrm{com}}^{*}\rangle$; Eq. (11)	*μ_lin_*; Eq. (9)	▽*_w_μ_lin_*; Eqs. (4) and (9)
	4	$\langle r_{\mathrm{ale}}^{*}\rangle$; Eq. (12)	*μ_lin_*; Eq. (9)	▽*_w_μ_lin_*; Eqs. (4) and (9)
Mixed linear	5	$\langle r_{\mathrm{lin}}^{*}\rangle$; Eq. (9)	*μ_lin_*; Eq. (9)	▽*_w_μ_lin_*; Eqs. (4) and (9)
	6	$\langle r_{\mathrm{out}}^{*}\rangle$; Eq. (10)	*μ_out_*; Eq. (10)	▽*_w_μ_lin_*; Eqs. (4) and (9)
	7	$\langle r_{\mathrm{com}}^{*}\rangle$; Eq. (11)	*μ_com_*; Eq. (11)	▽*_w_μ_lin_*; Eqs. (4) and (9)
	8	$\langle r_{\mathrm{ale}}^{*}\rangle$; Eq. (12)	*μ_ale_*; Eq. (12)	▽*_w_μ_lin_*; Eqs. (4) and (9)
Full gradient	9	$\langle r_{\mathrm{lin}}^{*}\rangle$; Eq. (9)	*μ_lin_*; Eq. (9)	▽*_w_μ_lin_*; Eqs. (4) and (9)
	10	$\langle r_{\mathrm{out}}^{*}\rangle$; Eq. (10)	*μ_out_*; Eq. (10)	▽*_w_μ_out_*; Eqs. (4) and (10)
	11	$\langle r_{\mathrm{com}}^{*}\rangle$; Eq. (11)	*μ_com_*; Eq. (11)	▽*_w_μ_com_*; Eqs. (4) and (11)
	12	$\langle r_{\mathrm{ale}}^{*}\rangle$; Eq. (12)	*μ_ale_*; Eq. (12)	▽*_w_μ_ale_*; Eqs. (4) and (12)
Shortest path	13	$\langle r_{\mathrm{lin}}^{*}\rangle$; Eq. (9)	*μ_lin_*; Eq. (9)	$\vec{\Phi }$; Eq. (16)
	14	$\langle r_{\mathrm{out}}^{*}\rangle$; Eq. (10)	*μ_out_*; Eq. (10)	$\vec{\Phi }$; Eq. (16)
	15	$\langle r_{\mathrm{com}}^{*}\rangle$; Eq. (11)	*μ_com_*; Eq. (11)	$\vec{\Phi }$; Eq. (16)
	16	$\langle r_{\mathrm{ale}}^{*}\rangle$; Eq. (12)	*μ_ale_*; Eq. (12)	$\vec{\Phi }$; Eq. (16)

The logic of these conditions is as follows: The 16 conditions are divided in sets of four, with the title indicated in the first column of this table. Every set includes all four types of reward model. In the first set, the value function is linear and the update rules assumes a gradient descent based on the linear value function. This set makes these assumptions despite the reward model not always being linear (Conditions 2–4). Because of the doubly linear assumptions, we call this set the Purely-linear conditions. In contrast, the second set assumes a value function matched to the reward models. However, the update rule continues to be linear and thus, we call this set the Mixed-linear conditions. Next is the set called the Full-gradient conditions. This is the only set respecting fully the reward models in both the value functions and the gradient-descend update rules. Finally, the Shortest-path conditions also have values functions respectful of rewards but use the Phi rule instead of the delta rule.

The main model in Aleem et al. (2020) corresponds to Condition 4.

### Methods for Computer Simulations

We must simulate Equations (1–4) to implement the delta rule and Equations (1–3), and (16) for the Phi rule. Combining these two sets of equations, we get respectively

$$\frac{d\vec{w}(t)}{dt}=\;\in_{\delta }m(t)\left(r^{*}(t)-\mu \left(\vec{u}(t):\vec{w}(t)\right)\right)\nabla_{w}\mu \left(\vec{u}(t):\vec{w}(t)\right),$$

(19) $$\frac{d\vec{w}(t)}{dt}=\;\in_{\Phi }m(t)\left(r^{*}(t)-\mu \left(\vec{u}(t):\vec{w}(t)\right)\right)\;\vec{\Phi }\left(\vec{w}(t):\vec{u}(t),r^{*}(t)\right).$$

We use possibly different *ε*_δ_ and *ε*_Φ_ to allow for a fair comparison between the convergence rates of the two processes, as explained in the “Standard Simulation Parameters” section. Equations (19) are stochastic differential equations (Aleem et al., 2020).

We simplify our simulations through a mean-field approximation of Equation (6):

$$\frac{d\vec{w}(t)}{dt}=\;\in_{\delta }\bar{m}\left(\vec{u}(t):{\vec{I}}_{m}\right)\;\left(r^{*}(t)-\mu \left(\vec{u}(t):\vec{w}(t)\right)\right)\nabla_{w}\mu \left(\vec{u}(t):\vec{w}(t)\right),$$

(20) $$\frac{d\vec{w}(t)}{dt}=\;\in_{\Phi }\bar{m}\left(\vec{u}(t):{\vec{I}}_{m}\right)\;\left(r^{*}(t)-\mu \left(\vec{u}(t):\vec{w}(t)\right)\right)\;\Phi \left(\vec{w}(t):\vec{u}(t),r^{*}(t)\right),$$

where $\bar{m}\left(\vec{u}(t):{\vec{I}}_{m}\right)$ is the mean motivation as a function of the sensory inputs $\vec{u}(t)$ and parametric on ${\vec{I}}_{m}$ (Aleem et al., 2020).

To approximate a solution to Equations (20), we must discretize time and sample, $\vec{u}$, *m*, and r* for every *t*. We do this discretization as follows:

$$\vec{w}(t_{k+1})=\vec{w}(t_{k})+\in_{\delta }^{'}\bar{m}\;(\vec{u}(t_{k+1}):{\vec{I}}_{m})\left(r^{*}(t_{k+1})-\mu \left(\vec{u}(t_{k+1}):\vec{w}(t_{k})\right)\right)\nabla_{w}\mu \left(\vec{u}(t_{k+1}):\vec{w}(t_{k})\right),$$

(21) $$\vec{w}(t_{k+1})=\vec{w}(t_{k})+\in_{\delta }^{'}\bar{m}\;(\vec{u}(t_{k+1}):{\vec{I}}_{m})\left(r^{*}(t_{k+1})-\mu \left(\vec{u}(t_{k+1}):\vec{w}(t_{k})\right)\right)\;\Phi \left(\vec{w}(t_{k}):\vec{u}(t_{k+1}),r^{*}(t_{k+1})\right),$$

where $\in_{\delta }^{'}=\;\in_{\delta }(t_{k+1}-t_{k})$ and $\in_{\Phi }^{'}=\;\in_{\Phi }(t_{k+1}-t_{k})$, with *t*_*k* +1_ − *t*_*k*_ being constant (for *k* = 0, 1, 2, …).

In this article, we compute *▽_w_μ* analytically. These gradients are relatively easy to compute, so we omit them here from the sake of space. As for the computation of $\vec{\Phi }$, we use the method of Marching Squares Algorithm to obtain the value isolines (Maple, 2003), and then apply Equations (14) and (15). We apply this algorithm to a 101 × 101 pixels approximation of the value function.

### Stochastic Sampling

To simulate Equations (21), one must sample $\vec{u}$ and r* stochastically from the probability distributions in Equation (5), and compute $\bar{m}\left(\vec{u}(t):{\vec{I}}_{m}\right)$ for use in Equations (20). We follow Aleem et al. (2020) and take five steps to simplify the sampling to make the simulations fast. See Aleem et al. (2020) for more details and justifications.

A. We did not simulate social “noise” by implementing explicitly $P\left({\vec{I}}_{u}|\vec{B}\right)$ and $P\left({\vec{I}}_{m}|\vec{B}\right)$, instead setting individual parameters by hand.
B. We split the individual parameters ${\vec{I}}_{u}$ into sensory related $\left({\vec{I}}_{s}\right)$ and reward related $\left({\vec{I}}_{r}\right)$:
  (22) $${\vec{I}}_{u}=\left[{\vec{I}}_{s},{\vec{I}}_{r}\right].$$
C. We made $\vec{u}$ two-dimensional. One component was visual balance (*u_b_*) and the other was visual complexity (*u_c_*), making
  $$\vec{u}=\left[u_{b},u_{c}\right],$$
  where 0 ≤ *u_b_*, *u_c_* ≤ 1, as per the definitions elsewhere (Aleem et al., 2017). Thus, while our model is amenable to a range of sensory inputs, we simplified it to the visual domain for illustrative purposes. Accordingly, *N* = 2 in Equation (11), and *u*_1_ = *u_b_* and *u*_2_ = *u_c_* in Equation (12) and Figure 1.
D. To be compatible with the two-dimensional $\vec{u}$ and so that all value functions have the same number of free parameters, we have set the number of free parameters in each model of reward to two. The models in Equations (10–12) have other parameters, namely, *α*_1_, *β*_1_, *α*_2_, *β*_2_, *α*_3_, and *β*_3_. However, we treat them as constants, with values specified in the “Standard Simulation Parameters” section.
E. We split the second term of Equation (5) as follows:
  (23) $$P\left(\left(\vec{u},r^{*}\right)|{\vec{I}}_{u}\right)=P\left(\vec{u}|{\vec{I}}_{s}\right)P\left(r^{*}|\vec{u},{\vec{I}}_{r}\right).$$

With these simplifications in hand, we followed Aleem et al. for the sampling of $\vec{u}$ through the first term of the right-hand side of Equation (23). We also followed them for the subsequent computation of $\bar{m}\left(\vec{u}(t):{\vec{I}}_{m}\right)$. For the sake of space, we refer the reader to their article (see their Equations 12, 13, 18, and 19).

Finally, we must specify how to sample r* through the second term of the right-hand side of Equation (23). We model $P\left(r^{*}|\vec{u},{\vec{I}}_{r}\right)$ as a Gaussian distribution with one of the means as in Equations (9–12):

(24) $$P\left(r_{x}^{*}|\vec{u}:{\vec{I}}_{r}\right)=\left[{\vec{w}}^{\left(x\right)},\vec{k}=\left[{\vec{k}}^{\left(x\right)},\sigma_{x}\right]\right]=\frac{1}{\sqrt{2\pi }\sigma_{x}}e^{-\frac{\left(r^{*}-\langle r_{x}^{*}\rangle \left(\vec{u}:{\vec{w}}^{\left(x\right)},{\vec{k}}^{\left(x\right)}\right)\right)}{2\sigma_{x}^{2}}},$$

where *x* ϵ {*lin*, *out*, *com*, *ale*}, and ${\vec{w}}^{\left(x\right)},{\vec{k}}^{\left(x\right)},$ and *σ_x_* > 0 are constant parameters.

### Summary of the Simulation Procedures

The simulations proceed with the following algorithm:

a. Suppose that at time *t_k_*, the vector of free parameters is $\vec{w}(t_{k})$.
b. Sample $\vec{u}(t_{k+1})=\left[u_{b}(t_{k+1}),u_{c}(t_{k+1})\right]$ from Equation (12) of Aleem et al. (2020).
c. Sample $\langle r_{x}^{*}t_{k+1}\rangle$ from Equation (24), with the definitions of $\langle r_{x}^{*}\rangle$ in Equations (9–12).
d. Compute $\bar{m}\left(\vec{u}(t_{k+1}):{\vec{I}}_{m}\right)$ from Equation (18) of Aleem et al. (2020).
e. Compute $\vec{w}(t_{k+1})$ from Equation (21).
f. Start the process again at Step a but at time *t*_*k* + 1_.

See Aleem et al. (2020) for more details on this algorithm.

All simulations were performed with code specially written in MATLAB.

### Standard Simulation Parameters

In this article, we report on simulations with different parameter sets to explore the various models. We have designated one of these sets as our standard set because the corresponding results capture the data in the literature reasonably well (Aleem et al., 2020). Table 2 shows the parameters of the standard simulations. Parameters for other simulations are indicated as appropriate in the Results (“Results” section).

**Table 2:** Standard set of parameters.

Parameter(s)	Equation(s)	Values
$\vec{w}(t_{0})$	(21)	[0, 0]
*ϵ_δ_*	(21)	0.01
*ϵ_Φ_*	(21)	0.007454
*t*_*k* +1_ − *t_k_*	(21)	1
$\left[{\vec{w}}^{\left(out\right)},\alpha_{1},\beta_{1},\sigma_{out}\right]$	(9) and (24)	[0.6, 0.9, 0.1414]
$\left[{\vec{w}}^{\left(lin\right)},\sigma_{lin}\right]$	(10) and (24)	[1.2, 1.8, 10, 1.5, 0.1414]
$\left[{\vec{w}}^{\left(com\right)},\alpha_{21},\beta_{21},\alpha_{22},\beta_{22},\sigma_{com}\right]$	(11) and (24)	[1.2, 1.8, 10, 0.6, 10, 0.9, 0.1414]
$\left[{\vec{w}}^{\left(out\right)},\alpha_{3},\beta_{3},\sigma_{out}\right]$	(12) and (24)	[0.6, 1, 0.75, 0.1, 0.1414]

A parameter in this table merits special discussion, namely, *ϵ*_Φ_ = 0.007454. We chose this value to make the comparison of the convergence rates of the gradient delta rule and the Phi rule fair. Changes of $\vec{w}$ in both rules are proportional to *δ* times a vector indicating the direction of change. In the delta rule, the vector is *▽_w_μ* whereas in the Phi rule, the vector is $\vec{\Phi }$, with the latter being a unit vector, while the former possibly having variable magnitudes. To make the convergence rate fair, we wanted to make the magnitudes of *ϵ_δ_* × *▽_w_μ* comparable to the magnitudes of $\in_{\Phi }\times \vec{\Phi }$. We did so by obtaining the root mean square of the magnitude of *ϵ_δ_* × ▽*_w_μ_lin_*, which is 0.7454, and thus because *ϵ_δ_* = 0.01, we got *ϵ_Φ_* = 0.007454.

### Statistics to Test the Hypotheses

All analyses comparing these statistics across the stimulated conditions (Table 1) used one-way ANOVA followed by *post-hoc* two-sided *t*-tests. For each of the Conditions 1–16, we ran 10 repetitions with 1,000,000 iterations each.

The statistics used to test our hypotheses (“Hypotheses Tested in This Article” sections) are summarized in Table 3.

**Table 3:** Statistics used to test the hypotheses.

Symbol	Title	Hypotheses
*τ_c_*	Time of convergence	I–III and V
*τ_r_*	Time of recovery from fluctuation errors	I–III and V
*δ_f_*	Final regret	I–III and V
${\vec{w}}_{f}$	Final free parameters of the value function	VI
ρ¬	Deviation from straightness	IV

To start the estimation of these statistics, we began by obtaining the fully motivated value curve obtained for the most common stimulus, namely, $\vec{u}=[u_{b},u_{c}]=[0.5,0.5]$ (Aleem et al., 2020). This curve was $v^{*}(t)=\mu \left(\left[0.5,0.5]:\vec{w}(t\right)\right)$ [Equation (1)].

From this curve, we first estimated *τ_c_* as the number of iterations needed for v*(*t*) to reach 90% of the median of v*(*t*) during the last 100,000 iterations. Similarly, we used these 100,000 iterations of v*(*t*) to estimate *τ_r_*. This statistic was important because it determined how many iterations we had to consider to avoid correlated measurements of the variable under consideration. We estimated this statistic through the autocorrelation coefficient (Park, 2018), by measuring when it decayed to 0.1 and setting that time to *τ_r_*. We also tested whether *τ_c_* and *τ_r_* were correlated across all the conditions in Table 1. For this purpose, we used the robust Kendall’s *τ* correlation coefficient (Bonett and Wright, 2000).

With *τ_r_* in hand, we could proceed to measure the values of *δ_f_* and ${\vec{w}}_{f}$. To measure these statistics, we obtained the medians of *δ* and $\vec{w}$ respectively over the last 2 × *τ_r_* iterations of each simulation. By considering 2 × *τ_r_* iterations, we could make sure to have two sets of temporally independent measurements.

Finally, to measure ρ¬ we first obtained the phase diagram of the free parameters, that is, *w*_2_(*t*) vs. *w*_1_(*t*). As we will see in the “Results” section, we can model the initial portion of this plot in our simulations as the straight line *w*_2_(*t*) = *kw*_1_(*t*), where *k* > 0 is a constant, and *w*_1_(*t*), *w*_2_(*t*) > 0 for *t* > 0. We estimated this line by robust linear regression, using M-estimation with Tukey’s biweight function (Rousseeuw and Leroy, 2003) from all the iterations such that *t* ≤ *τ_c_*. The plot then sometimes deviated from this line, meandering from it a certain distance. To measure the deviation from straightness, we used three points: $\vec{w}(t_{0})$ (Table 2), ${\vec{w}}_{f}=[w_{f,1},w_{f,2}]$ (Table 3), and the point $\vec{w}(t_{n})$ in the line *w*_2_(*t*) = *kw*_1_(*t*) that was nearest to ${\vec{w}}_{f}$. From these points, we defined the deviation from straightness as the signed ratio of the distance from ${\vec{w}}_{f}$ to $\vec{w}(t_{n})$ to the distance from $\vec{w}(t_{n})$ to $\vec{w}(t_{0})$. The sign was positive if ${\vec{w}}_{f}$ was above the line and negative otherwise. This definition using a signed ratio was valid because the denominator was always positive with our simulations. Straightforward calculus and algebra showed

(25) $$\rho \neg =\frac{w_{f,2}-kw_{f,1}}{kw_{f,2}+w_{f,1}}.$$

Consequently, −∞≺ρ¬≤∞, with ρ¬=0 if and only if ${\vec{w}}_{f}$ was on the initial straight line. Highly positive ρ¬ meant that final aesthetic preferences had a strong bias towards complexity, whereas highly negative ρ¬ meant a strong balance bias.

To test Hypothesis VI, we ran a one-way ANOVA on each of the components of ${\vec{w}}_{f}$ over the 10 repetitions of each condition.

## Results

### Limitations of the Purely-Linear Conditions

If the brain acquires aesthetic biases through reinforcement learning, neural circuitries implementing suitable value functions and update rules are necessary for good performance. We propose that good value functions and update rules depend on the statistics of sensory inputs, motivations, and rewards. Here, we focus on the latter. We do so because learning to predict rewards is the goal of the learning process. We thus built several models of reward, one linear and three nonlinear, and tested the learning performance of four value functions and three types of update rules (Table 1).

**Figure 2:**
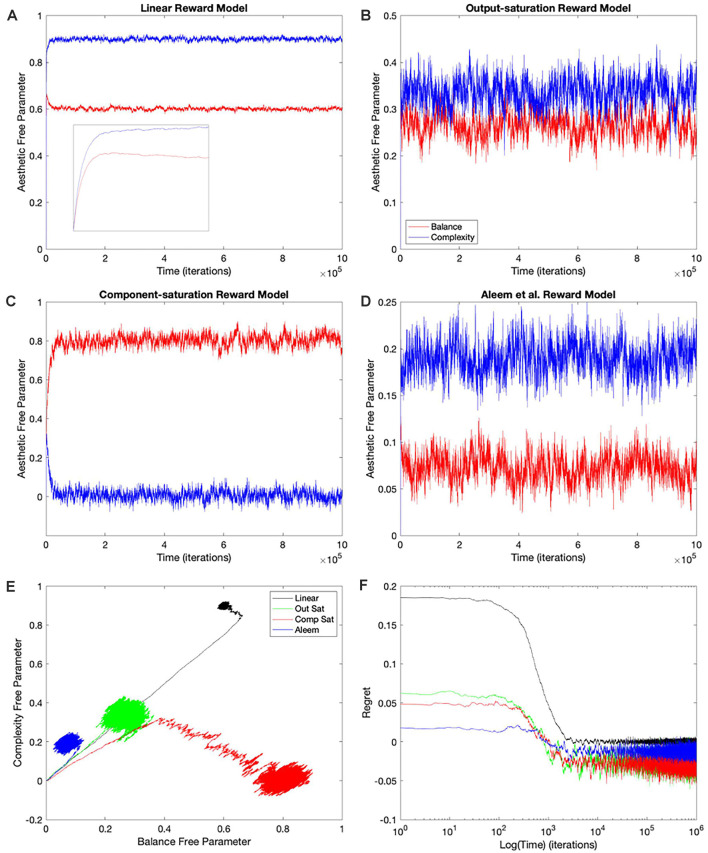
Dynamics of the free parameters of the value function for the purely-linear conditions (Table 1). **(A–D)** Linear, Output-saturation, Component-saturation, and Aleem et al.’s reward models respectively. Red and blue lines correspond respectively to free parameters related to balance and complexity in the sensory inputs. The inset in **(A)** provides details of the early dynamics (first 10,000 iterations). **(E)** Phase diagrams. **(F)** Time dependence of regrets, smoothed with a 500-iterations moving average. This figure indicates that linear value functions and update rules yield poor learning performance when the reward models are nonlinear. For example, regrets are significantly negative (overestimation of reward) for all nonlinear reward models (Panel **F**).

The simplest and thus, the most used combination of value function and update rule for reinforcement learning in the brain is purely linear (Conditions 1–4 in Table 1). Is learning performance with this combination good even when facing nonlinear reward models? Figure 2 shows the results of simulations with this combination of value function and update rule. The figure includes the temporal progression of free parameters, their phase diagrams, and errors in the prediction of reward. The simulations are performed for the four types of reward model studied in this article.

In all the simulations, the free parameters rose rapidly initially (Figures 2A–D). This rise occurred because these free parameters correlated positively with reward (Aleem et al., 2020). However, for some conditions, the fast rise ended and one of the free parameters started to fall as the other continued to climb (Figures 2A,C). This apparent competition of free parameters eventually stopped and the simulations reached steady state. We will address the reason for this apparent competition in the “Failing Hypotheses: How Stochasticity Helps and Shapes Learning” section. The apparent competition was especially evident in the phase diagrams (Figure 2E). With apparent competition, the phase diagram first seemed to rise linearly and then meandered away from the straight line (see the Linear and the Aleem et al.’s reward models in Figure 2E).

The apparent competition between the free parameters was not reflected in the temporal dependence of values. They rose and reached a steady state without any inflection points (Figure 7 of Aleem et al. —the results here were similar; data not shown). The lack-of-inflection point result is not surprising, because as shown in the “Update Rules for Value Functions” section, although free parameters do not statistically reach a unique fixed point, values do (Aleem et al., 2020). Furthermore, the delta rule used in these simulations tends to minimize value regret in a gradient-decent manner (“Update Rules for Value Functions” section; Appendix: Minimization of Regret Under Optimal Value Functions and the Delta Rule). Hence, values monotonically approach optimal results, even if the free parameters display strange behaviors.

**Figure 3:**
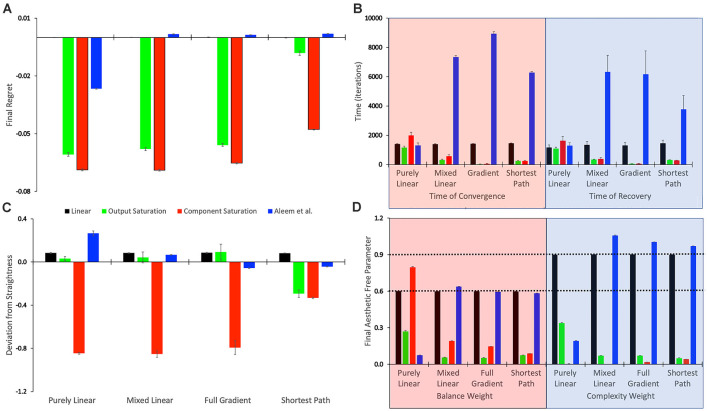
Statistics of the tested conditions (Table 1). **(A)** Final regret. **(B)** Times of convergence and of recovery from fluctuation errors. **(C)** Deviation from straightness. **(D)** Final free parameters of the value functions. each of these statistics was measured 10 times for each of the reward models, with the means and standard errors displayed. Color bins indicate the different reward models (colors matched to Figures 2E,F). We group the 16 conditions in sets of four according to the experimental conditions (Table 1). These sets were the purely linear, mixed linear, full gradient, and shortest path. The sets appear twice in Panel **(B)**, for time of convergence (transparent red) and time of recovery from fluctuation errors (transparent blue). Similarly, the sets appear twice in Panel **(D)**, for balance (transparent red) and complexity (transparent blue) free parameters. The dotted horizontal lines indicate the parameters of the linear reward models.

**Figure 4:**
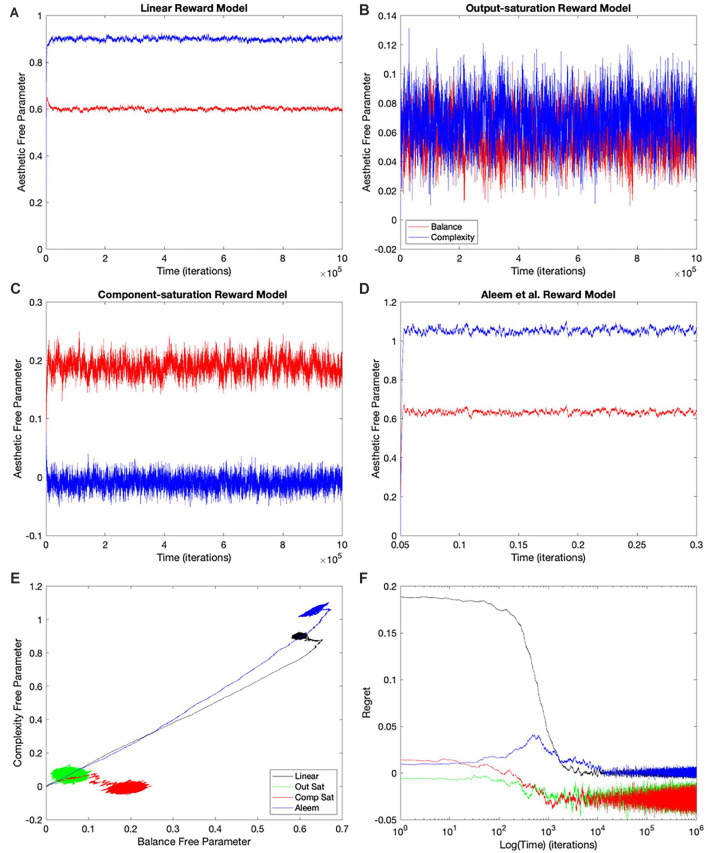
Dynamics of the free parameters of the value function for the Mixed-linear conditions (Table 1). The conventions for this figure are the same as those for Figure 2. The results here are qualitatively like those in Figure 2; but some significant quantitative differences are readily apparent. Thepanels and conventions for this figure are the same as those for Figure 2.

**Figure 5:**
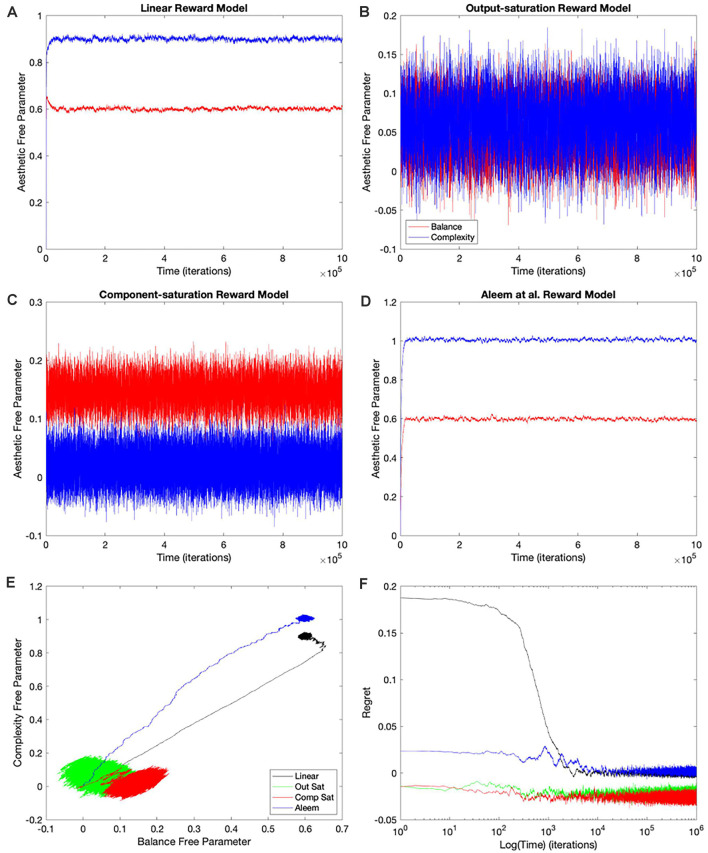
Dynamics of the free parameters of the value function for the full-gradient conditions (Table 1). The conventions for this figure are the same as those for Figure 2. The results here are qualitatively like those in Figure 4; with only minor quantitative differences being easily observable. Thepanels and conventions for this figure are the same as those for Figure 2.

**Figure 6:**
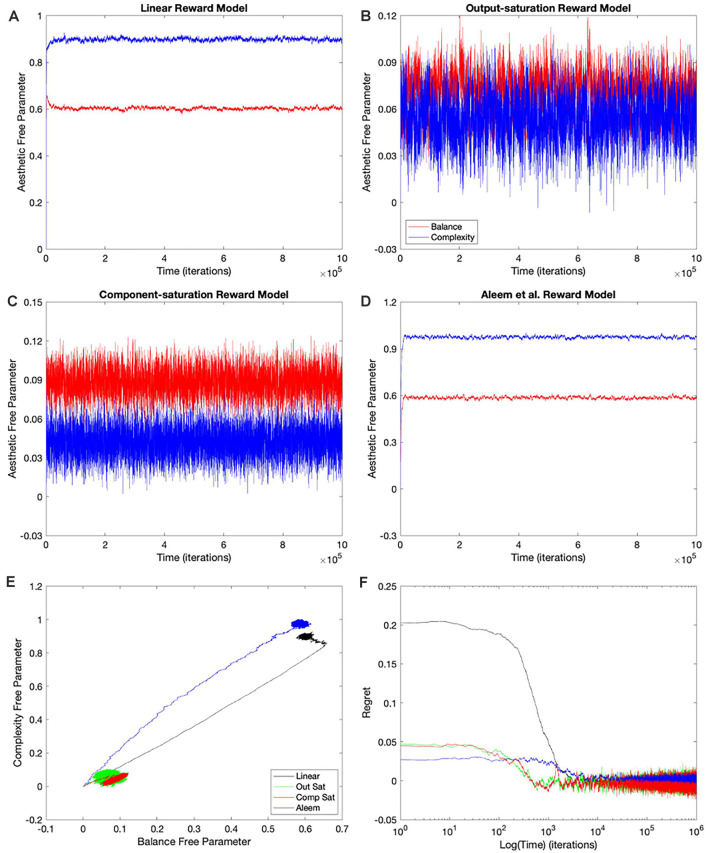
Dynamics of the free parameters of the value function for the shortest-path (Phi Rule) conditions (Table 1). The conventions for this figure are the same as those for Figure 2. The free-parameter and phase-diagram results here are qualitatively like those in Figure 5; with minor apparent quantitative differences (Panels **A–E**). However, although the changes in the free-parameter curves are subtle, the improvement of the regrets are dramatic (Panel **F**).

**Figure 7:**
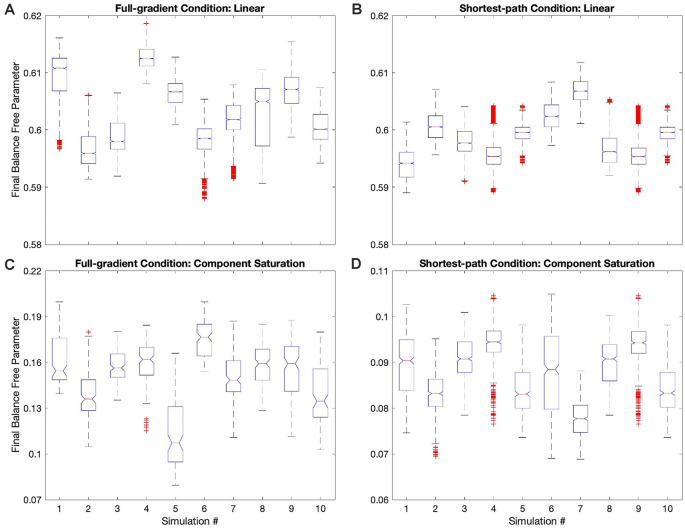
Box plots of the final balance free parameter in each of the simulations of four conditions in Table 1. **(A)** Full-gradient delta rule with linear value function. **(B)** Shortest-path (Phi) rule with linear value function. **(C)** Full-gradient delta rule with Component-saturation value function. **(D)** Shortest-path (Phi) rule with Component-saturation value function. Each box plot contains the 10 simulations of the indicated condition. On each box, the central mark is the median, and the edges of the box are the 25th and 75th percentiles. The whiskers are extended to the most extreme data points that are not considered outliers, with those being plotted individually using red “+” symbols. Box plots include notches for the comparison of the median values. Two medians are significantly different at the 5% significance level if their intervals, represented by notches, do not overlap. In all these four examples, the median final balance free parameters varied significantly across the simulations.

Going back to the temporal plots, we almost always observed the free parameter of complexity being larger than that of balance in these simulations (Figures 2A,B,D). This advantage of complexity was not surprising. We set up the simulations such that the fixed parameters of complexity made it contribute more to reward than those of balance (Table 2). However, when the reward model used the Component–saturation nonlinearity, the opposite happened and balance won (Figures 2C,E). The plots of regret provided further evidence of the inadequacy of the Purely-linear conditions (Figure 2F). Only when the reward model was linear did the final regret stay near zero. For all nonlinear reward models, the final regret was significantly negative (overestimation of reward).

To quantify the performance of the Purely-linear conditions, we measured the five statistics indicated in Table 3. The first statistic, regret (*δ_f_*), indicated the overall error of the estimation of reward after the learning process had converged. Next, the time of convergence (*τ_c_*), estimated how long the learning process took to converge. A related statistic was *τ_r_*, which captured how long the learning process took to recover from a fluctuation error. In turn, the deviation from straightness (ρ¬ captured how directly the learning trajectory went to the final goal. Finally, we measured with ${\vec{w}}_{f}$ where the free parameters converged at the end of the simulation. These results are summarized in Figure 3.

As expected, the magnitudes of the final regrets were large when using the Purely-linear strategy with nonlinear reward models (Figure 3A). These regrets were negative (overestimation of reward). However, the regrets were not significantly different from zero for the linear reward model (*δ_f_* = −0.0001 ± 0.0001; mean ± standard error). Although the regrets were statistically different from each other (one-way ANOVA and *post-hoc* two-sided *t*-test), the times of convergence were roughly similar (*τ_c_* ≈ 1,400 iterations—Figure 3B). Likewise, the times of recovery of fluctuation errors were roughly comparable (*τ_r_* ≈ 1,200 iterations—Figure 3B). The times of recovery exhibited a strong positive correlation with the times of convergence across all the conditions of Table 1 (Figure 3B; Kendall’s *τ* = 0.93, *p* < 4 × 10^−10^). As for deviations from straightness, all but the Output-saturation reward yielded results significantly different from zero (Figure 3C). These deviations were positive (advantage to complexity) or negative (advantage to balance). Interestingly, the Purely-linear simulations deviated from zero even for the linear reward model (ρ¬ = 0.084 ± 0.004; *t* = 20.0, 9 *d. f.*, *p* < 1 × 10^−8^).

In conclusion, the simulations with the Purely-linear conditions rule out Hypothesis I (“Hypotheses Tested in This Article” sections). This hypothesis fails because of the non-zero final regrets observed despite using a linear value function. We also rule out Hypothesis IV, since the linear value function yielded curved trajectories for all but the Output-saturation reward model. Finally, the inversion of complexity and balance preferences in Figure 2C rules out Hypothesis VII. On Table 2, the parameters of complexity are larger than those of balance, making the sensitivity coefficients for the former larger than for the latter. Therefore, Hypothesis VII would predict complexity preferences to be always larger than those for balance.

### Simulations With the Mixed-Linear Conditions

Using a linear value function tends to lead to a poor learning performance when the reward model is nonlinear (Figures 2, 3), but does the outcome improve when one uses the appropriate nonlinear value function? Would we observe an improvement even if the update rule continues to be linear? To answer these questions, we performed the simulations for the Mixed-linear conditions (Table 1). Figure 4 shows the results of these simulations. These results are important because they address the Hypotheses II and III in the “Hypotheses Tested in This Article” sections.

A comparison of Figure 4 with Figure 2 revealed that the Purely and Mixed-linear conditions yielded qualitatively, but not quantitively, similar learning performances. The ordering of the free-parameter curves (Figures 4A–D) were largely similar for the two sets of conditions. So were the shapes of the phase diagrams (Figure 4E) and the regret behaviors (Figure 4F). This similarity included the surprising “error” in ordering for the behavior of the Component-saturation curves (Figure 4C). However, the final free parameters were smaller for the Saturation reward models and larger for Aleem et al. in the Mixed-linear conditions. In addition, the magnitudes of final regrets were smaller. Figure 3A quantifies the improvement of the final regret for the Aleem reward model. In contrast, both Saturation reward models did not show statistically significant changes in terms of regret. Surprisingly, however, the time of convergence became faster for the Saturation reward models (*τ_c_* ≈ 430 iterations) and slower for Aleem et al. reward *τ_c_* = 7,300 ± 100 iterations (Figure 3B). The times of recovery from fluctuation errors exhibited similar results (Figure 3B). Finally, the magnitude of deviations from straightness fell for the Aleem et al.’s reward model (Figure 3C).

We conclude that Hypothesis II is also not valid. It fails because the Mixed-linear conditions include the Output-saturation value function, which yields no improvement in the final regret. Moreover, we can reject Hypothesis III because the magnitude of final regret for the Aleem et al. value function is smaller than for the Linear one. However, the slowness of both convergence and recovery from fluctuation errors with the Aleem et al. value function is predicted by the second part of Hypothesis III. Similarly, the straightness of the trajectory with the Output-saturation value function supports the second part of Hypothesis IV. The curvatures with the Component-saturation and Aleem et al. value functions also do so.

### Simulations With the Full-Gradient Conditions

Why does the Mixed-linear conditions not improve the performance with the Output and Component-saturation reward models despite using the appropriate value functions? Is the failure due to the use of an inappropriate (linear) update rule? A simple way to answer these questions is to implement the gradient update fully in the simulations. This is exactly what the Full-gradient conditions of Table 1 aim to achieve. The results of the simulations with these conditions appear in Figure 5.

The learning performances in Figure 5 were like those in Figure 4. The only apparent changes in Figure 5 were more noise in the Saturation conditions, and closer final free parameters of balance and complexity for Component Saturation (Figure 5C). However, inspection of the statistics in Figure 3 revealed small but significant improvements with the Full-gradient conditions. For example, the final regret improved slightly for the Aleem et al. function from *δ_f_* = 0.0017 ± 0.0001 to *δ_f_* = 0.0013 ± 0.0001 (*t* = 2.26, 18 *d. f*., *p* < 0.04). The statistics also revealed faster times to convergence for the Saturation value functions (*τ_c_* ≈ 40 iterations; *t* = 4.64, 18 *d. f*., *p* < 3 × 10^−4^ for Output Saturation). The times of recovery from fluctuation errors again exhibited similar results. In terms of deviation from straightness, the notable result was the change of sign for the Aleem et al. value function. The deviation from straightness changed from ρ¬=0.066±0.002 to ρ¬=−0.057±0.003.

Consequently, employing the appropriate update rules in a gradient-based delta-rule model helps the learning performance, but the effects are minor.

### Improved Performance With the Shortest-Path (Phi Rule) Conditions

Even with the Full-gradient conditions, the learning performance is still wanting (Figure 3), especially for nonlinear reward models. Figure 1 provides a possible explanation for the deficiency of performance based on gradient-based delta rules. The gradient is taken at the position of the vector of free parameters. Therefore, the direction of the gradient is generally blind to the curvatures of the isolines of the value function (Figures 1C,D). We have then proposed a new update rule that bypasses this deficiency of the gradient-based delta rule. If the value function is known, a calculation can be performed of the direction minimizing the path from the vector of free parameters to the target isoline (Figures 1E,F). We have called this update rule the Shortest-path or Phi rule (“Update Rules for Value Functions” section). The results of the simulations with this new rule appear in Figure 6.

Figure 6 shows that the Shortest-path (Phi) update rule produces superior performance when compared to the Full-gradient delta rule (Figure 5). The best evidence for the improved performance is that the magnitudes of final regrets are smaller with the Phi rule than with the delta rule (red curves in Figures 5C, 6C). This is confirmed in Figure 3A; especially for the Saturation conditions. The magnitude of the deviation from straightness also fell for the Component-saturation condition (Figure 3C; *t* = 7.23, 18 *d. f*., *p* < 2 × 10^−6^). Furthermore, this deviation fell for the Aleem et al. value functions (*t* = 3.76, 18 *d. f*., *p* < 0.002). Finally, the time of convergence fell for Aleem et al. value function from *τ_c_* = 8,900 ± 100 to *τ_c_* = 6,290 ± 70 iterations (Figure 3). The time of recovery from fluctuation errors also exhibited similar results (Figure 3B).

In conclusion, the Shortest-path (Phi) rule leads to superior learning performance as compared to the delta rule. However, the performance is not perfect. Imperfections include the small but non-zero regrets, small but significant deviations from straightness, and the relatively slow convergence and recovery for the Aleem et al. value function. Hence, the results reject Hypothesis V that states that the Phi Rule should yield no regret, and fast convergence and recovery from fluctuation errors.

### Non-uniqueness of the Learned Free Parameters

Hypothesis VI predicts that regardless of the update rule for the value function, the value reaches a unique fixed point (albeit only statistically), but the free parameters do not. The reason for the lack of uniqueness is that many parameter combinations yield the same value (isolines in Figure 1). To test this non-uniqueness hypothesis, we have inspected the statistics of the final free parameters of the simulations. The statistics appear in Figures 3D, 7; which shows box plots for each of the 10 individual simulations in some of the conditions in Table 1.

The statistics in Figure 3D initially suggested that at least for some conditions, the free parameters converged statistically to a unique fixed point. For example, the linear value function, which we repeated over the four sets of conditions, yielded final estimated parameters indistinguishable from those of the reward function (dotted horizontal lines in Figure 3D). The estimated value-function parameters for the delta rule (*N* = 30) were *w*_*f*, 1_ = 0.5999 ± 0.0007 and *w*_*f*, 2_ = 0.9005 ± 0.0008. In turn, the estimated value-function parameters for the Phi rule (*N* = 10) were *w*_*f*, 1_ = 0.598 ± 0.001 and *w*_*f*, 2_ = 0.900 ± 0.001. These estimated value-function parameters were statistically the same as the reward parameters, which were ${\vec{w}}^{\left(lin\right)}=[0.6,0.9]$ (Table 2).

However, closer inspection of the data reveals that the free parameters do not converge statistically to a unique fixed point. Figure 7 illustrates this conclusion with four examples of conditions in Table 1. (However, the conclusion applies to all conditions—data not shown). In these examples, we focus on the final balance free parameter and break down the results into the 10 simulations that give rise to each bin of Figure 3D. The first example to comment here is the one described in the last paragraph). As the Figure 7A shows, although the final balance free parameter hovers close to 0.6 (≈2.5% variation), the outcomes of the different simulations are not statistically homogeneous (one-way ANOVA, *F* = 5,960, 9 numerator d.f., 26,080 denominator d.f. *p* < 10^−15^). This inhomogeneity is not due to autocorrelations of the value signal (“Statistics to Test the Hypotheses” section). In addition, the inhomogeneity is applicable if one uses the Phi instead of the delta rule (Figure 7B, ≈1.5% variation, *p* < 10^−15^). Finally, the inhomogeneity remains if the value function is nonlinear. Figures 7C,D illustrate this latter conclusion for the Component-saturation value function, using the delta and Phi rules respectively. The respective variations are approximately 25% and 15%. And the one-way ANOVA tests yield *p* < 10^−15^ for both cases.

In closing, we cannot strictly speaking reject Hypothesis VI, because the free parameters do not converge statistically to a unique fixed point. However, the breakdown of uniqueness is less than expected from Figure 1. For example, the variation of final balance free parameters is small, being less than 2.5% for the linear value function. The small variation and non-uniqueness of convergence, leads us to define the concept of region (instead of point) of convergence.

### Failing Hypotheses: How Stochasticity Helps and Shapes Learning

The sections “Limitations of the Purely-linear Conditions” to “Non-uniqueness of the Learned Free Parameters” sections ruled out the hypotheses raised in the “Hypotheses Tested in This Article” sections, except possibly for Hypothesis VI, whose test nevertheless yielded a surprising result. Why did those hypotheses fail? In the “Hypotheses Tested in This Article” sections, we mentioned that we formulated the hypotheses by disregarding the stochastic nature of the learning process. In this section, we show that the stochasticity of the process has more effect on the learning outcome than expected.

**Figure 8:**
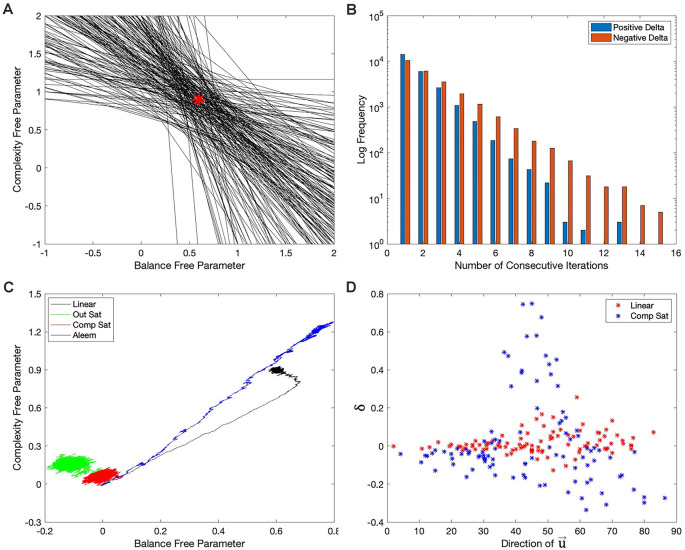
Explanations for the failures of the hypotheses in the “Hypotheses Tested in This Article” sections. **(A)** First 200 target isolines in the simulations of Figure 7A. The red star indicates the parameters of the model of reward (Table 2). The red star lies in the middle of the small region defined by the intersection of the target isolines. **(B)** Distribution of the number of consecutive iterations spent before (blue) and beyond (red) the target isolines in the last 100,000 iterations of the simulations of Figure 2B. The free parameters take longer to recover when they are beyond the target isoline than after it. **(C)** Phase diagram similar to Figure 2E but with the motivation function set to 1. The phase diagrams continue to exhibit curvatures, except possibly for that with the Aleem et al.’s reward model. **(D)** Comparison of 100 consecutive iterations (iteration 1,401 to iteration 1,500) with the linear (Figure 2A) and Component-saturation (Figure 2C) rewards models. The results with the Linear model (red dots) exhibit little correlation between *δ* and the direction of $\vec{u}$. But a strong, complex correlation is evident for the Component-saturation model (blue).

To understand why stochasticity led to the rejection of all but one of the hypotheses raised by Figure 1, we dove deeper into the surviving hypothesis. Although the final free parameters did not predictably exhibit uniqueness according to Hypothesis VI, their variation was much less than expected (Figure 7). Why was the variation so small? To answer this question, consider initially the linear value function (Figure 1A). The expectation of large variation of final free parameters was due to every point on the target isoline giving the same prediction of reward. However, because we drew the sensory inputs and rewards randomly across iterations, the slopes and intercepts of the isolines changed. Consequently, the target isoline changed across iterations. But the intersections of the target isolines crossed in a small region around the fixed parameters of the reward model (Figure 8A). Therefore, the variations of the final free parameters were smaller than we would expect by only considering the non-stochastic process (Figure 1A). The same low-variation result applied to the nonlinear value functions (data not shown). The stochasticity of the learning process thus helped improve the acquired final free parameters.

Similarly, the stochasticity helped explain the failure of the other hypotheses. Hypothesis I failed because of the non-zero final regrets observed despite using a linear value function when the reward model was nonlinear (Figures 2F, 3A). Consider for example the nonlinear Output-saturation model in Figure 1B. In this model, the contour plot also consisted of parallel straight isolines. When the learning converged around the right solution, the stochastic process sometimes took the free parameters beyond the target isoline and sometimes before it. As shown in Figure 1B, the gradient was larger before than beyond that isoline. The larger gradient caused the recovery to be faster for the former kind of error. Thus, the value overestimated reward on average, that is, the free parameters spent more time recovering beyond the target isoline than before it. The consequence was that when the regret is positive, it stayed so for fewer iterations than when it was negative (Figure 8B). The regret was thus negative on average (Figures 2F, 3A). Similar regret reasons helped explain why Hypotheses II, III, and V failed (details not discussed here for the sake of brevity).

Stochasticity also explained why we could reject Hypothesis IV. We ruled it out because the linear value function yielded curved trajectories for all but the Output-saturation reward model (Figure 2E). An initial hypothesis for what caused these curved trajectories was the motivation function Equation (1). Aleem et al. (2020) showed that making this function a constant eliminated the curved trajectory in their model. However, their model corresponded only to Condition four in Table 1, so we could not be sure that their result would apply to all the conditions in Figure 2E. When we probed this possibility by setting the motivation to a constant, we generally did not eliminate the curvatures of the trajectories in that figure. The only exception was for the Aleem et al.’s reward model (Figure 8C).

Further investigation revealed that the reason for the curvatures was due to something more fundamental and again, related to the stochasticity of the learning process. The argument explaining the reason was mathematical. Taking the mean-field approximation of Equation (19) (Chaikin and Lubensky, 2007) and neglecting the probabilistic variations of *m* (because it does not matter for the curvatures) we get

$$\frac{d\vec{w}(t)}{dt}=\;\in_{\delta }m\;\left({\langle r^{*}(t)-\nabla_{w}\mu \left(\vec{u}(t):\vec{w}(t)\right)\rangle }_{r^{*},\vec{u}}-{\langle \mu \left(\vec{u}(t):\vec{w}(t)\right)\nabla_{w}\mu \left(\vec{u}(t):\vec{w}(t)\right)\rangle }_{\vec{u}}\right),$$

(26) $$\frac{d\vec{w}(t)}{dt}=\;\in_{\Phi }m\;\left({\langle r^{*}(t)-\vec{\Phi }\left(\vec{w}(t):\vec{u}(t),r^{*}(t)\right)\rangle }_{r^{*},\vec{u}}-{\langle \mu \left(\vec{u}(t):\vec{w}(t)\right)\;\vec{\Phi }\left(\vec{w}(t):\vec{u}(t),r^{*}(t)\right)\rangle }_{\vec{u}}\right),$$

The term inside the parenthesis in this equation is the subtraction of two averages over r* and $\vec{u}$. These averages are like those in Equation (17), using the sampling of r* and $\vec{u}$ at every *t*. Consider the situation in which the value $\mu \left(\vec{u}(t):\vec{w}(t)\right)$ is a poor predictor of the reward r*(*t*). If the value underestimates the reward grossly, then the first average dominates the dynamics. If instead the value overestimates the reward grossly, then the second average dominates. Either way, the dominance gives rise to the initial, straight trajectory of the simulations (Figures 2E, 4E, 5E, 6E). When the simulations approach the region of convergence, both averages begin to contribute simultaneously to the slower, more random trajectory. Now, the first but not the second average depend on the statistics of r*. Hence, the initial and final trajectories are generally in different directions, giving rise to the curvatures.

Finally, stochasticity was also at the core of why Hypothesis VII failed. The inversion of complexity and balance free parameters in Figures 2C, Figure 4C, 5C, 6C ruled out Hypothesis VII. For this inversion to occur, the right-hand side of Equation (4) had to push the balance free parameters upward faster than the complexity ones. The function *δ* in Equation (4) was identical for the balance and complexity components of the vector $\vec{w}$. Similarly, $\nabla_{w}\mu \left(\vec{u}(t):\vec{w}(t)\right)$ did not depend on reward and thus, could not differentiate the importance of balance and complexity. Consequently, because $\nabla_{w}\mu \left(\vec{u}(t):\vec{w}(t)\right)$ depended only on $\vec{u}$, the explanation for why the balance free parameter grew more than the complexity one had to rely on the correlation between *δ* and $\vec{u}$. Did certain directions of $\vec{u}$ coincide with larger δ? Figure 8D demonstrated the correlation between *δ* and $\vec{u}$ with a sector of 100 points in the simulation giving rise to Figures 2A,C (This sector was from Iteration 1,401 to Iteration 1,500, but other sectors and other Computer Saturation simulations yielded similar results). The *δ* in the simulations with the Linear reward model was not strongly correlated with the direction of $\vec{u}$. However, the Component-saturation model yielded larger positive *δ* than the Linear model at low angles of $\vec{u}$ (closer to the balance axis). Moreover, for the most part, the Component-saturation model yielded negative δ, specially at the larger angles, that is, closer to complexity. Therefore, Figure 8D confirmed the correlation between *δ* and $\vec{u}$. This correlation was such that the Component-saturation model yielded statistically larger balance free parameters than complexity ones. Details of why the Component-saturation model exhibited the correlation seen in Figure 8D had to do with the specific shape of the nonlinearity and the statistics of $\vec{u}$. We left these details out of this paper for the sake of brevity.

## Discussion

An increasingly large number of neuroimaging studies have allowed us to begin understanding the basic neural circuitries underlying the computation of aesthetic biases in the brain (Brown et al., 2011). These circuitries are suggestive of computational mechanisms for the learning of these biases as a set of decision values. Their acquisition would take the form of reinforcement learning gated by internal mechanisms of motivation. Accordingly, a recent theoretical framework for the learning of aesthetic biases followed these computational mechanisms (Aleem et al., 2019, 2020). A model based on that framework could account for interesting features of human aesthetic biases. These features included individuality (Nelson and Morrison, 2005; Brown and Dissanayake, 2009; Silvia et al., 2009), cultural predispositions (Masuda et al., 2008; Park and Huang, 2010; Senzaki et al., 2014), stochastic dynamics of learning and aesthetic biases (Grzywacz and de Juan, 2003; Pouget et al., 2013; Aleem et al., 2020), and the peak-shift effect (Ramachandran and Hirstein, 1999; Costa and Corazza, 2006; Aleem et al., 2020). However, despite the success in explaining these features, a potential major weakness of the model in Aleem et al. (2020) was the linearity of the value function used to predict reward. Such an assumption of linearity is often made in reinforcement-learning models of brain function (Kaelbling et al., 1996; Sutton and Barto, 2018). In this research, we probe what would mean to relax this assumption. In this section, we discuss the effect of relaxing linearity on regret (“Minimization of Regret” section), learning rate (“Efficiency of Learning” and “Phi Versus Delta Rules” sections), and qualitative errors (“Does the Brain Use Ecological Value Functions?” section).

### Minimization of Regret

The learning performance exhibited significant regret (error) when using a linear value function to try to predict rewards arising from a nonlinear model. Others have proposed nonlinear value functions (Chung et al., 2018), methods to deal these functions (Xu et al., 2007; Gu et al., 2016; Osband et al., 2016), or their approximators (Tesauro, 2005; Kober et al., 2013; Mahadevan et al., 2013). Here, we attempted to develop optimal nonlinear value functions. The result was exciting because it told us that the optimal nonlinear value function related directly to the statistics of reward in a predictable manner [Equation (8)]. However, incorporating the optimal nonlinear value function helped with some nonlinear reward models but not others. We had expected better performance with these value functions when using the delta rule. Our expectation was due to the mathematical demonstration of the minimization of regret, even with nonlinear value functions. How did we explain this unmet expectation? The expectation of optimization came from a process of gradient descent implemented by the delta rule (Sutton and Barto, 2018). That the regret did not go to zero could have meant that a local minimum different from the global one trapped the gradient descent (Beck, 2017). Such traps might occur for some nonlinearities but not others.

However, the specific type of stochasticity in our models made it unlikely that their learning processes normally stopped at local minima. The stochastic mechanism arising from the probabilistic sampling of sensory stimuli, rewards, and motivations caused the target isolines to vary. The variation likely helped the free parameters to approach their optimal values (Figures 7A,C). This is not surprising because stochasticity often helps optimization processes (Metropolis et al., 1953; Kirkpatrick et al., 1983; Spall, 2003). But for our models, the interaction between stochasticity and the nonlinearities could also cause important errors. Even if the simulation succeeded in reaching exactly a target isoline, the next instant would produce a new one. At this new instant, the vector of free parameters could be before or beyond the new target isoline. The rate of recovery in these two conditions were different because of the model nonlinearity. Consequently, on average, the solution was not optimal, because of the interaction between stochasticity and the nonlinearities of the models. Errors of various forms of stochastic optimization have been described in other studies (Ingber, 1993; Shen et al., 2020). For example, errors could arise if the sampling were not truly stochastic. This could happen to some degree if predictions based on prior learning or motivational factors affected the sampling (Janis and Mann, 1977; Frey, 1986; Schulz-Hardt et al., 2000).

On the other hand, if the learning process occasionally stopped at local minima because of nonlinearities of value functions, it might explain a surprising result from the history of art. An analysis of the statistics of art across the Renaissance and Baroque revealed phase transitions in some measures (Correa-Herran et al., 2020). Another such abrupt transition was observed in a study of the changes in fractal dimension and Shannon entropy in Western paintings (Mather, 2018). The discussion by Correa-Herran et al. (2020) pointed out the essential components of such phase transitions. These components had to be nonlinear interactions between the basic components of a system, which was under the influence of changing external conditions. Correa-Herran et al. (2020) proposed that the basic elements were the values associated with different aesthetic variables. Hence, our proposed use of nonlinear value functions is compatible with the ideas of Correa-Herran et al. (2020). Following their proposal, our nonlinear value functions would generate nonlinear mutual influence between artists learning from each other (Aleem et al., 2019; Correa-Herran et al., 2020). In turn, according to Correa-Herran et al. (2020), the changing external conditions were due to the social pressure to innovate (Barnett, 1953). Such a pressure could come from the desire to increase realism during the Renaissance (Janson et al., 1997). More pressure came from the competition among artists to gain the favor of patrons (Chambers, 1970).

### Efficiency of Learning

An implication of the delta rule is that it tends to maximize the rate of learning convergence for the linear value function (Aleem et al., 2020). Under these conditions, the rate of recovery from fluctuation errors after convergence is also maximal. Therefore, these conditions should implement a highly efficient learning process, albeit with some caveats (Zomaya, 2006; Sutton and Barto, 2018). In contrast, for nonlinear value functions, the delta rule is not expected to lead to efficient learning in general (“Hypotheses Tested in This Article” sections). We thus expected the nonlinear value functions to lead to relatively slow convergence and recovery with the Full-gradient conditions. This expectation did not materialize for the Saturation conditions. We also expected the Shortest-path Phi rule to overcome these deficiencies of the gradient-based delta rule. The Phi rule does so by going directly to the optimal point on the target isoline. But again, this expectation for the Phi rule failed for the Saturation conditions.

How can we explain the failures of the expectations for efficiency of the learning rates of convergence and recovery? Here, we will focus on the time of convergence because its strong correlation with the time of recovery makes the answers similar. As discussed after Equation (26), the time of convergence is dominated by two factors: First, we must consider how far the free parameters must travel to reach the slower, more stochastic portion of the learning trajectory. This phase of the trajectory is reached when the two averages in Equation (26) become similar. Second, we must consider the speed of movement of the free parameters during the early, “straight” portion of the trajectory. This speed depends on the largest average of Equation (26). Hence, three factors may influence this speed. They are the gradient of the value function, the distance from the nearest point on the target isoline, and the correlation between reward and the direction of the vector of motion. Because these factors vary across value functions, the factors modulate the different efficiencies of the learning rates.

These two factors explain the various apparent efficiencies of the time of convergence. For example, the short phase-diagram trajectories of the Saturation conditions explain their fast convergence. In contrast, the long trajectories for the Linear and Aleem et al. conditions help explain their slow convergence. However, for these conditions, the speed of movement of the vector of free parameters during the early, “straight” portion of the trajectory also matters. The phase-diagram trajectories for the Linear and Aleem et al. conditions are almost as long. But the latter converges much more slowly than the former. This slow convergence for the Phi rule provides further evidence against Hypothesis V (“Hypotheses Tested in this Article” and “Failing Hypotheses: How Stochasticity Helps and Shapes Learning” sections).

### Phi vs. Delta Rules

The importance of the delta rule arguably derives from its simplicity of implementation, low computational cost, and differential-equation form (Widrow and Hoff, 1960; Stone, 1986). However, we argue here that the delta rule may do poorly when applied to some nonlinear value functions. In those situations, the gradient used in the rule has a non-optimal direction (Figures 1C,D). Alternatives could include gradient-free algorithms, but they do not tend to have the simple and differential forms (Kirkpatrick et al., 1983; Kennedy and Eberhart, 2001; Conn et al., 2009; Mockus, 2012). We thus proposed an alternate differential-equation-based rule that overcomes this deficiency. The new rule (called Phi or Shortest Path) does not estimate the direction of descent based on the gradient at the location of the vector of free parameters. Instead, the new rule uses holistic knowledge of the nonlinear value function to set the direction toward the optimal point on the target isoline. This holistic rule leads to better regret performance. Furthermore, because of its differential form, the Phi rule allows for a simple implementation as the delta rule.

However, the Phi rule has an important disadvantage when compared to the delta rule. The holistic implementation of the Phi rule is bound to make it computationally expensive and consequently, slow. In our implementations, the simulations with the Phi rule conditions were about five times slower that those with the delta rule. But we did not attempt to optimize our implementation of the Phi rule. The main step in such an optimization would be to find efficient algorithms to obtain the isolines of the value function. We used a standard implementation of the Marching Squares algorithm (Maple, 2003), but faster versions exist (Ho et al., 2005; Garrido et al., 2006). We also applied the algorithm to a 101 × 101 pixels approximation of the value function and perhaps a coarser approximation would be enough. In addition, we could have used other algorithms that are faster for isoline calculations (Yanchang and Junde, 2001). Finally, the holistic isoline computation is parallelizable (Selikhov, 1997; Belikov and Semenov, 2000; Huang, 2001; Dong et al., 2011), making it imminently efficient for brain-network computations.

### Does the Brain Implement Nonlinear Value Functions?

The brain has been often argued to linearize what would otherwise be nonlinear input dependencies (Yu and Lewis, 1989; Bernander et al., 1994; Ermentrout, 1998). Such linearization would allow the brain to map conceptual or perceptual dimensions using linear functions. For example, Naselaris et al. (2011) performed successful neuroimaging on many conceptual and perceptual dimensions often assuming such linearization. These authors’ results on linearization have been confirmed by other studies (Qiao et al., 2019). Moreover, linear value functions account for some forms of reinforcement learning in the basal ganglia (Schultz et al., 1997; Hollerman and Schultz, 1998; Schultz, 2015; Sutton and Barto, 2018). Hence, such value functions may sometimes provide a simpler, suitable model for neurobiological or psychological value updating than the models considered in this article.

However, two lines of argument suggest that this linearization argument is only an approximation that is not always valid. The first line is that some of the results above have been disputed. For example, the compressive spatial summation in human cortex (Kay et al., 2013) has challenged the unchecked applicability of the Naselaris et al.’s (2011) conclusions. This challenge is compatible with the neural representation of stimulus features becoming increasingly nonlinear as one moves along the sensory pathway (Holdgraf et al., 2017). Further limitation of assuming linearization is the nonlinear processing at high-in-the-hierarchy levels of the brain (Andrzejak et al., 2001; Faure and Korn, 2001; Freeman and Vitiello, 2006; Afraimovich et al., 2011). Finally, although some linear models for reinforcement learning in the basal ganglia are good enough, this process is decidedly nonlinear (Frank and Claus, 2006; Hsu et al., 2009; Niv et al., 2012).

Even more important is the argument that many empirically determined value functions in the brain are often nonlinear. An example for this argument in the visual domain comes from psychophysical studies of preference for complexity. The visual-complexity value function in humans is highly nonlinear, lying on an inverted “U” curve, with people liking moderate amounts of complexity (Berlyne, 1971; Aitken, 1974; Nicki and Moss, 1975; Saklofske, 1975; Imamoglu, 2000). Another nonlinear value function for the human visual system is indicated by the saturation relationship between preference and the number of the axes of symmetry in an image (Wu and Chen, 2020). Examples of nonlinear value functions in non-visual sensory modalities also exist. In the auditory system, for instance, the preference for a piece of music is a saturating function of the familiarity with the piece (Szpunar et al., 2004). Relatedly, preference for music has a U-shape dependence on harmonic surprise (Miles, 2018). And even when one leaves the pure sensory domain into social value, value functions are nonlinear. For example, the tendency of humans to adjust values to social conformity by reinforcement learning has a nonlinear dependence on mean social value (Klucharev et al., 2009).

The implications of the brain employing nonlinear value functions in many situations is important. As stated above, using linear value functions would often be good enough. The learning process would always converge because even if the brain erroneously assumes a linear value function, the process minimizes a positive functional (Aleem et al., 2020). And the convergence can occasionally be faster for erroneous linear value functions than for correct nonlinear ones. However, the price that the brain would be pay is large systematic regrets with erroneous linear value functions. Some degree of regret is unavoidable in the learning of aesthetic value because of the stochasticity of the process. But our results show that the brain can minimize regret in a statistical sense by choosing the appropriate value function. Therefore, by choosing to implement nonlinear value functions in many situations, the brain seems to be prioritizing the minimization of regret over the ease of computation.

### Does the Brain Use Ecological Value Functions?

Because the Phi rule requires holistic knowledge of the value function, one must ask how would the brain know what the value function is. An answer to this question is that the brain has a bank of socially and ecologically important value functions. Another answer is that the brain uses a single, multidimensional value function, capturing social and ecological values. The brain may develop such value functions through evolutionary pressure. This proposal echoes ecological and evolutionary ideas for sensory function (Field, 1987; Atick and Redlich, 1992; Grzywacz and de Juan, 2003). Alternatively, the brain could build ecological value functions through developmental and learning mechanisms. Again, this would be akin to the developmental models for optimal receptive fields in the sensory systems of the brain (MacKay and Miller, 1990; Miller, 1994; Burgi and Grzywacz, 1998). And this would be akin to learning new brain representations for familiar objects in adult life (Tarr, 1995; Weinberger, 1995; Booth and Rolls, 1998). Thus, if variables like balance, complexity, and symmetry have evolutionary importance, then the brain would develop dedicated circuitry, facilitating their computation and assignment of value. Such a dedicated circuitry would make sense because the optimal value function depends directly on the external statistics of reward [Equation (8)]. This link between the ease of dedicated computation and aesthetic value is the premise of the Processing Fluency theory (Reber et al., 2004; Aleem et al., 2017; Correa-Herran et al., 2020). The work here and elsewhere suggests that humans learn individually to weigh the various parameters of the ecological value functions (Aleem et al., 2019, 2020). This conclusion suggests that studying the statistics of reward may be as important as investigating the statistics of natural stimuli (Field, 1987; Ruderman and Bialek, 1994; Balboa et al., 2001; Balboa and Grzywacz, 2003).

However, the hypothetical use of ecological value functions implies a couple of limitations in the computation of aesthetic biases. One limitation would be the inability to learn new values outside the set provided by ecological pressures. The alternative would be to use general value functions that could capture both the ecological ones and some that may not be ecological. Examples of such general value functions were introduced elsewhere (Konidaris and Osentoski, 2008; Sutton et al., 2011; Schaul et al., 2015). Another limitation of using just ecological value functions is the error that they would make when a sensory stimulus does not fit their expectations. Using the wrong value function increases the magnitude of regret in the learning process. However, even when the value functions are right and optimal, quantitative and qualitative errors do occur. Errors like these and others are observed after reinforcement learning in the brain (O’Reilly and McClelland, 1994; Clouse, 1997; Niv, 2009; Gold et al., 2012; Dabney et al., 2020). Therefore, these kinds of errors may be unavoidable. The best that one can hope is to make important errors as small as possible. The important errors are not those of free parameters but of value, that is, of the estimation of reward. Value functions and update rules optimized for social and ecological constraints may thus be ideal for the learning of aesthetic biases.

## Data Availability Statement

The original contributions presented in the study are included in the article, further inquiries can be directed to the corresponding author.

## Author Contributions

NG developed the theoretical framework and its equations, carried out the mathematical analyses, computer simulations, statistical analysis of the results, and wrote the manuscript. 

## Conflict of Interest

The author declares that the research was conducted in the absence of any commercial or financial relationships that could be construed as a potential conflict of interest.

## Appendices

### Optimal Value Function

#### Claim 1

The expected least-squares error of the prediction of fully motivated reward by Equations (1–3), (5) and (6) is minimized by

(27) $$\mu_{opt}(\vec{u}:\vec{w},\vec{k})=\langle r^{*}\rangle \left(\vec{u}:{\vec{I}}_{u}=\left[\vec{w},\vec{k}\right]\right),$$

where $\langle r^{*}\rangle \left(\vec{u}:{\vec{I}}_{u}=\left[\vec{w},\vec{k}\right]\right)$ indicates the mean of *r** given the sampled sensory inputs, and the free $\left(\vec{w}\right)$ and constant $\left(\vec{k}\right)$ parameters of the value function.

#### Proof

We start from the expected least-squares error in Equation (8), namely,

(28) $$E=\underset{\vec{u},r^{*},m}{∭}P(\vec{u},r^{*})P(m|\vec{u}){\left(m\left(\mu (\vec{u})-r^{*}\right)\right)}^{2},$$

where we drop both the dependence on *t* and the parameters for the sake of conciseness. This equation indicates that the error is a functional of $\mu (\vec{u}(t))$. To calculate the optimal function, we fix $\vec{u}(t),$ and calculate

(29) $$\mu_{opt}(\vec{u})=\arg\min_{\mu^{*}}\iint_{r^{*},m}P\left(r^{*}|\vec{u}\right)P\left(m|\vec{u}\right){\left(m(\mu^{*}-r^{*})\right)}^{2}.$$

To find this minimum, we differentiate the integrals by *μ** and set the result to 0, yielding

$${∭}_{r^{*},m}P\left(r^{*}|\vec{u}\right)P\left(m|\vec{u}\right)m^{2}\left(\mu_{opt}-r^{*}\right)=0,\;\mu_{opt}\langle m^{2}\rangle (\vec{u})-\langle r^{*}\rangle (\vec{u})\langle m^{2}\rangle (\vec{u})=0,$$

where 〈〉 $\left(\vec{u}\right)$ indicates average given $\vec{u}$. This last equation proves our claim.

#### Comments on Claim 1

• The implication of Equation (27) is to tell us the optimal value function in the least-squares sense.
• Consequently, to find out what this function is, one must know the statistics of reward given the sensory stimuli.
• This conclusion suggests that studying the statistics of reward may be as important as investigating the statistics of natural stimuli.

### Minimization of Regret Under Optimal Value Functions and the Delta Rule

#### Claim 2

If for every τ there is a *t* > τ such that *m(t)* > 0, then the learning process minimizes

(30) $$E(\vec{w})=\langle m(t)\left(r^{*}(t)-\mu \left(\right)\right)\rangle$$

where ${\langle \rangle }_{t}$ stands for time average.

#### Proof

The gradient of *E* with respect to the components of $\vec{w}$ obeys

$$\nabla_{w}E(\vec{w})\propto -{\langle m(t)\left(r^{*}(t)-\mu \left(\vec{u}(t):\vec{w}(t)\right)\right)\nabla_{w}\mu \left(\vec{u}(t):\vec{w}(t)\right)\rangle }_{t},\;\nabla_{w}E(\vec{w})\propto -{\langle r(t)-v(t)\nabla_{w}\mu \left(\vec{u}(t):\vec{w}(t)\right)\rangle }_{t},$$

or

(31) $$\nabla_{w}E(\vec{w})\propto -{\langle \delta (t)\nabla_{w}\mu \left(\vec{u}(t):\vec{w}(t)\right)\rangle }_{t}$$

Hence, the process governed by Equation (4) minimizes $E(\vec{w})$ by performing a gradient descent (Strutz, 2016).

#### Comments on Claim 2

• The minimization of $E(\vec{w})$ with respect to the components of $\vec{w}$ in Equation (30) implies that $\mu (\vec{u}(t):\vec{w}(t))$ becomes statistically close to *r**(*t*). Equivalently, $v(t)=m(t)\mu \left(\vec{u}(t):\vec{w}(t)\right)$ becomes statistically close to *r*(*t*) = *m*(*t*) *r**(*t*). Therefore, the process optimizes value by making it as close as possible to reward.
• However, *ν*(*t*) may not converge exactly to *r*(*t*); “Minimization of Regret” section.
• The requirement that for every τ there is a *t* > τ such that *m*(*t*) > 0 is necessary to give the process enough time to reach optimization. If *m*(*t*) = 0 for every *t* > τ, then the learning process freezes after τ as shown by Equations (1–4).

### Perpendicularity Condition Under the Phi Rule

#### Claim 3

If one uses the Phi rule [Equations (13–16)] to update reinforcement learning, then Equation (18) holds.

#### Proof

The Phi rule calls for finding the point in the target isoline $\left({\vec{w}}_{opt}\right)$ that is closest to the vector of free parameters $\left(\vec{w}\right)$. We do this by using the Lagrange-multiplier method (Bertsekas, 1982). We thus build the Lagrangian function

(32) $$ℒ({\vec{w}}_{opt},\lambda )={\left({\vec{w}}_{opt},\vec{w}\right)}^{2}-\lambda \left(\mu (\vec{u}:\vec{w})-r^{*}\right),$$

where the first term of the right-hand side is the square of the distance between ${\vec{w}}_{opt}$ and $\vec{w}$, and the second term is the constraint of the target isoline $\left(\mu (\vec{u}:\vec{w})-r^{*}=0\right)$ times the multiplier *λ*. To minimize the distance, we must find the minimum of the Lagrangian function with respect to both ${\vec{w}}_{opt}$ and *λ*. We find this minimum by calculating the respective partial derivatives and setting them to zero:

$$\nabla_{{\vec{w}}_{opt}}ℒ({\vec{w}}_{opt},\lambda )=2\left({\vec{w}}_{opt}-\vec{w}\right)-\lambda \nabla_{{\vec{w}}_{opt}\mu }(\vec{u}:\vec{w})=0,$$

$$\nabla_{\lambda }ℒ\left({\vec{w}}_{opt},\lambda \right)=r^{*}-\mu (\vec{u}:\vec{w})=0,$$

which yields

(33) $${\vec{w}}_{opt}-\vec{w}=\frac{\lambda }{2}\nabla_{{\vec{w}}_{opt}}\mu (\vec{u}:\vec{w}),$$

(34) $$\mu (\vec{u}:\vec{w})=r^{*}.$$

These equations prove our claim.

#### Comments on Claim 3

• The meaning of Equations (33) and (34) is straightforward: Begin with the target isoline [Equation (34)] and find the points in it whose gradients are parallel to the line connecting $\vec{w}$ to ${\vec{w}}_{opt}$.
• These gradients are perpendicular to the isoline. Hence, we must calculate the directions perpendicular to the target isoline and find those that are parallel to the line connecting $\vec{w}$ to ${\vec{w}}_{opt}$.
• Sometimes, we may have multiple such directions, but this situation is rare.

## References

[B1] Afraimovich V. Young T. Muezzinoglu M. K. Rabinovich M. I. (2011). Nonlinear dynamics of emotion-cognition interaction: when emotion does not destroy cognition? Bull. Math. Biol. 73, 266–284. 10.1007/s11538-010-9572-x 20821062 PMC3208426

[B2] Aitken P. (1974). Judgments of pleasingness and interestingness as functions of visual complexity. J. Exp. Psychol. 103, 240–244. 10.1037/h0036787

[B3] Aleem H. Correa-Herran I. Grzywacz N. M. (2017). Inferring master painters’ esthetic biases from the statistics of portraits. Front. Hum. Neurosci. 11:94. 10.3389/2017.00094 28337133 PMC5343217

[B4] Aleem H. Correa-Herran I. Grzywacz N. M. (2020). A theoretical framework for how we learn aesthetic values. Front. Hum. Neurosci. 14:345. 10.3389/2020.00345 33061898 PMC7518219

[B5] Aleem H. Pombo M. Correa-Herran I. Grzywacz N. M. (2019). “Is beauty in the eye of the beholder or an objective truth? A neuroscientific answer,” in Mobile Brain-Body Imaging and the Neuroscience of Art, Innovation and Creativity, eds Contreras-Vidal J. Robleto D. Cruz-Garza J. G. Azorin J. M. Nam C. S. (Cham: Springer International Publishing), 101–110.

[B6] Andrzejak R. G. Lehnertz K. Mormann F. Rieke C. David P. Elger C. E. (2001). Indications of nonlinear deterministic and finite-dimensional structures in time series of brain electrical activity: dependence on recording region and brain state. Phys. Rev. E Stat. Nonlin. Soft. Matter Phys. 64:061907. 10.1103/PhysRevE.64.061907 11736210

[B7] Atick J. J. Redlich A. N. (1992). What does the retina know about natural scenes? Neural Comput. 4, 196–210. 10.1162/neco.1992.4.2.196

[B8] Balboa R. M. Grzywacz N. M. (2003). Power spectra and distribution of contrasts of natural images from different habitats. Vis. Res. 43, 2527–2537. 10.1016/s0042-6989(03)00471-1 13129540

[B9] Balboa R. M. Tyler C. W. Grzywacz N. M. (2001). Occlusions contribute to scaling in natural images. Vis. Res. 41, 955–964. 10.1016/s0042-6989(00)00302-3 11248280

[B10] Barnett H. G. (1953). Innovation: The Basis of Cultural Change. New York, NY: McGraw-Hill.

[B11] Beck A. (2017). First-Order Methods in Optimization. Philadelphia, PA: Society for Industrial and Applied Mathematics.

[B12] Belikov V. V. Semenov A. Y. (2000). Non-sibsonian interpolation on arbitrary system of points in euclidean space and adaptive isolines generation. Appl. Num. Math. 32, 371–387. 10.1016/s0168-9274(99)00058-6

[B13] Berlyne D. E. (1971). Aesthetics and Psychobiology. Cambridge, MA: Harvard University Press.

[B14] Bernander O. Koch C. Douglas R. J. (1994). Amplification and linearization of distal synaptic input to cortical pyramidal cells. J. Neurophysiol. 72, 2743–2753. 10.1152/jn.1994.72.6.2743 7897486

[B15] Bertsekas D. P. (1982). Constrained Optimization and Lagrange Multiplier Methods. New York, NY: Academic Press.

[B16] Biederman I. Vessel E. A. (2006). Perceptual pleasure and the brain: a novel theory explains why the brain craves information and seeks it through the senses. Am. Sci. 94, 247–253. 10.1511/2006.59.247

[B17] Bonett D. G. Wright T. A. (2000). Sample size requirements for estimating pearson, kendall and spearman correlations. Psychometrika 65, 23–28. 10.1007/bf02294183

[B18] Booth M. C. Rolls E. T. (1998). View-invariant representations of familiar objects by neurons in the inferior temporal visual cortex. Cereb. Cortex 8, 510–523. 10.1093/cercor/8.6.510 9758214

[B19] Brown S. Dissanayake E. (2009). “The arts are more than aesthetics: neuroaesthetics as narrow aesthetics,” in Foundations and Frontiers in Aesthetics. Neuroaesthetics, eds M. Skov and O. Vartanian (Amityville, NY: Baywood Publishing Co.), 43–57.

[B20] Brown S. Gao X. Tisdelle L. Eickhoff S. B. Liotti M. (2011). Naturalizing aesthetics: brain areas for aesthetic appraisal across sensory modalities. NeuroImage 58, 250–258. 10.1016/j.neuroimage.2011.06.012 21699987 PMC8005853

[B21] Burgi P. Y. Grzywacz N. M. (1998). A biophysical model for the developmental time course of retinal orientation selectivity. Vis. Res. 38, 2787–2800. 10.1016/s0042-6989(97)00323-4 9775326

[B22] Chaikin P. M. Lubensky T. C. (2007). Principles of Condensed Matter Physics (4th print edition). Cambridge, CA: Cambridge University Press.

[B23] Chambers D. (1970). Patrons and Artists in the Italian Renaissance. London, UK: McMillan.

[B24] Chatterjee A. Vartanian O. (2014). Neuroaesthetics. Trends Cogn. Sci. 18, 370–375. 10.1016/j.tics.2014.03.003 24768244

[B25] Chung W. Nath S. Joseph A. White M. (2018). “Two-timescale networks for nonlinear value function approximation,” in Paper Presented at the International Conference on Learning Representations, New Orleans, LA, 1–32.

[B26] Clouse J. A. (1997). The Role of Training in Reinforcement Learning (Vol. 121). Amsterdam, Netherlands: North Holland.

[B27] Conn A. R. Scheinberg K. Vicente L. N. (2009). Introduction to Derivative-Free Optimization. Philadelphia, PA: SIAM.

[B28] Correa-Herran I. Aleem H. Grzywacz N. M. (2020). Evolution of neuroaesthetic variables in portraits paintings throughout the renaissance. Entropy 22:146. 10.3390/e22020146 33285921 PMC7516560

[B29] Costa M. Corazza L. (2006). Aesthetic phenomena as supernormal stimuli: the case of eye, lip, and lower-face size and roundness in artistic portraits. Perception 35, 229–246. 10.1068/p3449 16583768

[B30] Dabney W. Kurth-Nelson Z. Uchida N. Starkweather C. K. Hassabis D. Munos R.. (2020). A distributional code for value in dopamine-based reinforcement learning. Nature 577, 671–675. 10.1038/s41586-019-1924-6 31942076 PMC7476215

[B31] Dann C. Neumann G. Peters J. (2014). Policy evaluation with temporal differences: a survey and comparison. J. Mach. Learn. Res. 15, 809–883.

[B32] Dong L. Lu D. Li M. (2011). “Parallel algorithm of visualization of reservoir numerical simulation based on pebi grids,” in Paper Presented at the 2011 Fourth International Symposium on Parallel Architectures, Algorithms and Programming, Tianjin, China, 302–305.

[B33] Ermentrout B. (1998). Linearization of f-i curves by adaptation. Neural Comput. 10, 1721–1729. 10.1162/089976698300017106 9744894

[B34] Faure P. Korn H. (2001). Is there chaos in the brain? I. Concepts of nonlinear dynamics and methods of investigation. C. R. Acad. Sci. III 324, 773–793. 10.1016/s0764-4469(01)01377-4 11558325

[B35] Field D. J. (1987). Relations between the statistics of natural images and the response properties of cortical cells. J. Opt. Soc. Am. A 4, 2379–2394. 10.1364/josaa.4.002379 3430225

[B36] Filiz-Ozbay E. Ozbay E. Y. (2007). Auctions with anticipated regret: theory and experiment. Am. Econ. Rev. 97, 1407–1418. 10.1257/aer.97.4.1407

[B37] Frank M. J. Claus E. D. (2006). Anatomy of a decision: striato-orbitofrontal interactions in reinforcement learning, decision making, and reversal. Psychol. Rev. 113, 300–326. 10.1037/0033-295X.113.2.300 16637763

[B38] Freeman W. J. Vitiello G. (2006). Nonlinear brain dynamics as macroscopic manifestation of underlying many-body field dynamics. Phys. Life Rev. 3, 93–118. 10.1016/j.plrev.2006.02.001

[B39] Frey D. (1986). “Recent research on selective exposure to information,” in Advances in Experimental Social Psychology, ed Berkowitz L. (New York, NY: Academic Press), 41–80.

[B40] Garrido S. Moreno L. Abderrahim M. Martin F. (2006). “Path planning for mobile robot navigation using voronoi diagram and fast marching”, in Paper Presented at the Intelligent Robots and Systems, 2006 IEEE/RSJ International Conference (Beijing, China: IEEE). 10.1109/IROS.2006.282649

[B41] Gilbert D. T. Morewedge C. K. Risen J. L. Wilson T. D. (2004). Looking forward to looking backward: the misprediction of regret. Psychol. Sci. 15, 346–350. 10.1111/j.0956-7976.2004.00681.x 15102146

[B42] Gold J. M. Waltz J. A. Matveeva T. M. Kasanova Z. Strauss G. P. Herbener E. S.. (2012). Negative symptoms and the failure to represent the expected reward value of actions: behavioral and computational modeling evidence. Arch. Gen. Psychiatry 69, 129–138. 10.1001/archgenpsychiatry.2011.1269 22310503 PMC4406055

[B43] Grzywacz N. M. de Juan J. (2003). Sensory adaptation as kalman filtering: theory and illustration with contrast adaptation. Network 14, 465–482. 10.1088/0954-898x_14_3_305 12938767

[B44] Gu S. Lillicrap T. Sutskever I. Levine S. (2016). “Continuous deep q-learning with model-based acceleration,” in Paper Presented at the International Conference on Machine Learning, New York City, NY, USA, 2829–2838.

[B45] Ho C. C. Wu F. C. Chen B. Y. Chuang Y. Y. Ouhyoung M. (2005). Cubical Marching Squares: Adaptive Feature Preserving Surface Extraction From Volume Data (Vol. 24). Oxford, UK and Boston, USA: Blackwell Publishing, Inc.

[B46] Holdgraf C. R. Rieger J. W. Micheli C. Martin S. Knight R. T. Theunissen F. E. (2017). Encoding and decoding models in cognitive electrophysiology. Front. Syst. Neurosci. 11:61. 10.3389/fnsys.2017.00061 29018336 PMC5623038

[B47] Hollerman J. R. Schultz W. (1998). Dopamine neurons report an error in the temporal prediction of reward during learning. Nat. Neurosci. 1, 304–309. 10.1038/1124 10195164

[B48] Hsu M. Krajbich I. Zhao C. Camerer C. F. (2009). Neural response to reward anticipation under risk is nonlinear in probabilities. J. Neurosci. 29, 2231–2237. 10.1523/JNEUROSCI.5296-08.2009 19228976 PMC6666337

[B49] Huang G.-M. (2001). Isoline 3-d display and its parallel algorithm. Science of Surveying and Mapping, 26, 20–22.

[B50] Hudspeth A. J. Jessell T. M. Kandel E. R. Schwartz J. H. Siegelbaum S. A. Eds. (2013). Principles of Neural Science, 5th Edn. McGraw-Hill, Health Professions Division.

[B51] Iigaya K. Yi S. Wahle I. A. Tanwisuth K. O’Doherty J. P. (2020). Aesthetic preference for art emerges from a weighted integration over hierarchically structured visual features in the brain. biorxiv [Preprint]. 10.1101/2020.02.09.940353

[B52] Imamoglu Ç. (2000). Complexity, liking and familiarity: architecture and nonarchitecture turkish students’assessments of traditional and modern house facades. J. Environ. Psychol. 20, 5–16. 10.1006/jevp.1999.0155

[B53] Ingber L. (1993). Simulated annealing: practice versus theory. Math. Comput. Model. 18, 29–57. 10.1016/0895-7177(93)90204-c

[B54] Janis I. L. Mann L. (1977). Decision Making: A Psychological Analysis of Conflict, Choice, and Commitment. New York, NY: Free Press.

[B55] Janson H. W. Janson A. F. Marmor M. (1997). History of Art. London: Thames and Hudson.

[B56] Kaelbling L. P. Littman M. L. Moore A. W. (1996). Reinforcement learning: a survey. J. Artif. Intell. Res. 4, 237–285. 10.1613/jair.301

[B57] Kay K. N. Winawer J. Mezer A. Wandell B. A. (2013). Compressive spatial summation in human visual cortex. J. Neurophysiol. 110, 481–494. 10.1152/jn.00105.2013 23615546 PMC3727075

[B58] Kennedy J. Eberhart R. C. (2001). Swarm Intelligence. San Francisco, CA: Morgan Kaufmann

[B59] Kirkpatrick S. Gelatt C. D. Jr. Vecchi M. P. (1983). Optimization by simulated annealing. Science 220, 671–680. 10.1126/science.220.4598.671 17813860

[B60] Klucharev V. Hytönen K. Rijpkema M. Smidts A. Fernandez G. (2009). Reinforcement learning signal predicts social conformity. Neuron 61, 140–151. 10.1016/j.neuron.2008.11.027 19146819

[B61] Kober J. Bagnell J. A. Peters J. (2013). Reinforcement learning in robotics: a survey. Int. J. Robot. Res. 32, 1238–1274. 10.1177/0278364913495721

[B62] Konidaris G. Osentoski S. (2008). “Value function approximation in reinforcement learning using the fourier basis,” in Computer Science Department Faculty Publication Series, 101 (MA: University of Massachusetts Amherst), 1–11.

[B63] Kreps D. M. (1990). A Course in Microeconomic Theory. Princeton, NJ: Princeton University Press.

[B64] Lacey S. Hagtvedt H. Patrick V. M. Anderson A. Stilla R. Deshpande G.. (2011). Art for reward’s sake: visual art recruits the ventral striatum. NeuroImage 55, 420–433. 10.1016/j.neuroimage.2010.11.027 21111833 PMC3031763

[B65] Leder H. Nadal M. (2014). Ten years of a model of aesthetic appreciation and aesthetic judgments: the aesthetic episode-developments and challenges in empirical aesthetics. Br. J. Psychol. 105, 443–464. 10.1111/bjop.12084 25280118

[B66] MacKay D. J. C. Miller K. D. (1990). Analysis of linsker’s application of hebbian rules to linear networks. Netw. Comp. Neural Syst. 1, 257–297. 10.1088/0954-898x_1_3_001

[B67] Maei H. R. (2011). Gradient Temporal-Difference Learning Algorithms. Ph.D. Thesis. Edmonton, AB: University of Alberta.

[B68] Mahadevan S. Giguere S. Jacek N. (2013). “Basis adaptation for sparse nonlinear reinforcement learning,” in Paper Presented at the Association for the Advancement of Artificial Intelligence, Bellevue, Washington, USA, 654–660.

[B69] Mahmood A. R. Sutton R. S. (2015). “Off-policy learning based on weighted importance sampling with linear computational complexity,” in Paper Presented at the 31st Conference on Uncertainty in Artificial Intelligence, Amsterdam, Netherlands, 552–561.

[B70] Maple C. (2003). “Geometric design and space planning using the marching squares and marching cube algorithms,” in Proceedings of the International Conference on Geometric Modeling and Graphics, London, United Kingdom, 90–95.

[B71] Martindale C. (1984). The pleasures of thought: a theory of cognitive hedonics. J. Mind Behav. 5, 49–80.

[B72] Masuda T. Gonzalez R. Kwan L. Nisbett R. E. (2008). Culture and aesthetic preference: comparing the attention to context of east asians and americans. Pers. Soc. Psychol. Bull. 34, 1260–1275. 10.1177/0146167208320555 18678860

[B73] Mather G. (2018). Visual image statistics in the history of western art. Art Percept. 6, 97–115. 10.1163/22134913-20181092

[B74] Metropolis N. Rosenbluth A. W. Rosenbluth M. N. Teller A. H. Teller E. (1953). Equation of state calculations by fast computing machines. J. Chem. Phys. 21, 1087–1092. 10.1063/1.1699114 41342507

[B75] Miles S. A. (2018). The Relationship Between the Perception of Unexpected Harmonic Events and Preference in Music. Ph.D. Dissertation. Washington, DC: Georgetown University.

[B76] Miller K. D. (1994). A model for the development of simple cell receptive fields and the ordered arrangement of orientation columns through activity-dependent competition between on- and off-center inputs. J. Neurosci. 14, 409–441. 10.1523/JNEUROSCI.14-01-00409.1994 8283248 PMC6576834

[B77] Mockus J. (2012). Bayesian Approach to Global Optimization: Theory and Applications. Dordrecht, Netherlands: Kluwer Academic.

[B78] Nadal M. Chatterjee A. (2019). Neuroaesthetics and art’s diversity and universality. Wiley Interdiscip. Rev. Cogn. Sci. 10:e1487. 10.1002/wcs.1487 30485700

[B79] Naselaris T. Kay K. N. Nishimoto S. Gallant J. L. (2011). Encoding and decoding in fmri. NeuroImage 56, 400–410. 10.1016/j.neuroimage.2010.07.073 20691790 PMC3037423

[B80] Nelson L. D. Morrison E. L. (2005). The symptoms of resource scarcity: judgments of food and finances influence preferences for potential partners. Psychol. Sci. 16, 167–173. 10.1111/j.0956-7976.2005.00798.x 15686584

[B81] Nicki R. Moss V. (1975). Preference for non-representational art as a function of various measures of complexity. Can. J. Psychol. 29, 237–249. 10.1037/h0082029

[B82] Niv Y. (2009). Reinforcement learning in the brain. J. Math. Psychol. 53, 139–154. 10.1016/j.jmp.2008.12.005

[B83] Niv Y. Edlund J. A. Dayan P. O’Doherty J. P. (2012). Neural prediction errors reveal a risk-sensitive reinforcement-learning process in the human brain. J. Neurosci. 32, 551–562. 10.1523/JNEUROSCI.5498-10.2012 22238090 PMC6621075

[B84] O’Doherty J. P. Dayan P. Friston K. Critchley H. Dolan R. J. (2003). Temporal difference models and reward-related learning in the human brain. Neuron 38, 329–337. 10.1016/s0896-6273(03)00169-7 12718865

[B85] O’Reilly R. C. McClelland J. L. (1994). Hippocampal conjunctive encoding, storage, and recall: avoiding a trade-off. Hippocampus 4, 661–682. 10.1002/hipo.450040605 7704110

[B86] Osband I. Blundell C. Pritzel A. Van Roy B. (2016). “Deep exploration *via* bootstrapped DQN,” in Advances in Neural Information Processing Systems 29 (NIPS 2016), 4026–4034.

[B88] Park K. I. (2018). Fundamentals of Probability and Stochastic Processes with Applications to Communications. New York, NY: Springer.

[B87] Park D. C. Huang C.-M. (2010). Culture wires the brain: a cognitive neuroscience perspective. Perspect. Psychol. Sci. 5, 391–400. 10.1177/1745691610374591 22866061 PMC3409833

[B89] Pouget A. Beck J. M. Ma W. J. Latham P. E. (2013). Probabilistic brains: knowns and unknowns. Nat. Neurosci. 16, 1170–1178. 10.1038/nn.3495 23955561 PMC4487650

[B90] Qiao K. Chen J. Wang L. Zhang C. Zeng L. Tong L.. (2019). Category decoding of visual stimuli from human brain activity using a bidirectional recurrent neural network to simulate bidirectional information flows in human visual cortices. Front. Neurosci. 13:692. 10.3389/fnins.2019.00692 31354409 PMC6630063

[B91] Ramachandran V. S. Hirstein W. (1999). The science of art: a neurological theory of aesthetic experience. J. Conscious. Stud. 6, 15–51.

[B92] Reber R. Schwarz N. Winkielman P. (2004). Processing fluency and aesthetic pleasure: is beauty in the perceiver’s processing experience? Pers. Soc. Psychol. Rev. 8, 364–382. 10.1207/s15327957pspr0804_3 15582859

[B93] Riesz F. Szökefalvi-Nagy B. (1990). Functional Analysis. New York, NY: Dover Publications.

[B94] Rousseeuw P. J. Leroy A. M. (2003). Robust Regression and Outlier Detection. New York, NY: John Wiley & Sons.

[B95] Ruderman D. L. Bialek W. (1994). Statistics of natural images: scaling in the woods. Phys. Rev. Lett. 73, 814–817. 10.1103/PhysRevLett.73.814 10057546

[B96] Saklofske D. H. (1975). Visual aesthetic complexity, attractiveness and diversive exploration. Percept. Mot. Skills 41, 813–814. 10.2466/pms.1975.41.3.813 1215122

[B97] Saltelli A. Ratto M. Andres T. Campolongo F. Cariboni J. Gatelli D.. (2008). Global Sensitivity Analysis: The Primer. New York, NY: John Wiley & Sons.

[B98] Schaul T. Horgan D. Gregor K. Silver D. (2015). “Universal value function approximators,” in Paper Presented at the International Conference on Machine Learning, Lille, France, 1312–1320.

[B99] Schmidhuber J. (2010). Formal theory of creativity, fun, and intrinsic motivation. IEEE Trans. Auton. Ment. Dev. 2, 230–247. 10.1109/tamd.2010.2056368

[B100] Schultz W. (1998). Predictive reward signal of dopamine neurons. J. Neurophysiol. 80, 1–27. 10.1152/jn.1998.80.1.1 9658025

[B101] Schultz W. (2015). Neuronal reward and decision signals: from theories to data. Physiol. Rev. 95, 853–951. 10.1152/physrev.00023.2014 26109341 PMC4491543

[B102] Schultz W. (2016). Dopamine reward prediction error coding. Dialogues Clin. Neurosci. 18, 23–32. 10.31887/DCNS.2016.18.1/wschultz 27069377 PMC4826767

[B103] Schultz W. Dayan P. Montague P. R. (1997). A neural substrate of prediction and reward. Science 275, 1593–1599. 10.1126/science.275.5306.1593 9054347

[B104] Schulz-Hardt S. Frey D. Lüthgens C. Moscovici S. (2000). Biased information search in group decision making. J. Pers. Soc. Psychol. 78, 655–669. 10.1037//0022-3514.78.4.655 10794372

[B105] Selikhov A. (1997). Cellular Algorithm for Isoline Extraction From a 2d Image (Vol. 6). Joint Bulletin of the Novosibirsk Computer Center and the Institute of Informatics Systems. National Curriculum Council.

[B106] Senzaki S. Masuda T. Nand K. (2014). Holistic versus analytic expressions in artworks: cross-cultural differences and similarities in drawings and collages by canadian and japanese school-age children. J. Cross Cult. Psychol. 45, 1297–1316. 10.1177/0022022114537704

[B107] Shen W. Yang Z. Ying Y. Yuan X. (2020). Stability and optimization error of stochastic gradient descent for pairwise learning. Anal. Appl. 18, 887–927. 10.1142/s0219530519400062

[B108] Silvia P. J. Henson R. A. Templin J. L. (2009). Are the sources of interest the same for everyone? Using multilevel mixture models to explore individual differences in appraisal structures. Cogn. Emot. 23, 1389–1406. 10.1080/02699930902850528

[B109] Skov M. (2010). “The pleasure of art,” in Pleasures of the Brain, eds Kringelbach M. L. Berridge K. C. (New York, NY: Oxford University Press), 270–283.

[B110] Somasundaram J. Diecidue E. (2016). Regret theory and risk attitudes. J. Risk Uncertain. 55, 1–29. 10.1007/s11166-017-9268-9

[B111] Spall J. C. (2003). Introduction to Stochastic Search and Optimization: Estimation, Simulation, and Control. Hoboken, NJ: Wiley.

[B112] Stone G. O. (1986). “An analysis of the delta rule and the learning of statistical associations,” in Parallel Distributed Processing: Explorations in the Microstructure of Cognition, Vol. I, eds Rumelhart D. E. McClelland J. L. (Cambridge, MA: The MIT Press), 444–459.

[B113] Strutz T. (2016). Data Fitting and Uncertainty: A Practical Introduction to Weighted Least Squares and Beyond, 2nd Edn. Wiesbaden, Germany: Springer Vieweg.

[B114] Sutton R. S. Barto A. G. (2018). Reinforcement Learning: An Introduction. Cambridge, MA: The MIT Press.

[B115] Sutton R. S. Modayil J. Delp M. Degris T. Pilarski P. M. White A.. (2011). “Horde: a scalable real-time architecture for learning knowledge from unsupervised sensorimotor interaction,” in Paper Presented at the International Conference On Autonomous Agents and Multi-Agent Systems, Taipei, Taiwan, 761–768.

[B116] Szpunar K. K. Schellenberg E. G. Pliner P. (2004). Liking and memory for musical stimuli as a function of exposure. J. Exp. Psychol. Learn. Mem. Cogn. 30, 370–381. 10.1037/0278-7393.30.2.370 14979811

[B117] Tarr M. J. (1995). Rotating objects to recognize them: a case study on the role of viewpoint dependency in the recognition of three-dimensional objects. Psychon. Bull. Rev. 2, 55–82. 10.3758/BF03214412 24203590

[B118] Tesauro G. (2005). “Online resource allocation using decompositional reinforcement learning,” in Paper Presented at the Association for the Advancement of Artificial Intelligence, Pittsburgh, PA, 886–891.

[B119] Tsitsiklis J. N. Van Roy B. (1997). “Analysis of temporal-diffference learning with function approximation,” in Paper Presented at the Advances in Neural Information Processing Systems (NIPS), Vancouver, Canada, 1–32.

[B120] Van de Cruys S. Wagemans J. (2011). Putting reward in art: a tentative prediction error account of visual art. Iperception 2, 1035–1062. 10.1068/i0466aap 23145260 PMC3485793

[B121] Vartanian O. Skov M. (2014). Neural correlates of viewing paintings: evidence from a quantitative meta-analysis of functional magnetic resonance imaging data. Brain Cogn. 87, 52–56. 10.1016/j.bandc.2014.03.004 24704947

[B122] Vessel E. A. Rubin N. (2010). Beauty and the beholder: highly individual taste for abstract, but not real-world images. J. Vis. 10, 18.1–18.14. 10.1167/10.2.18 20462319 PMC3662030

[B123] Vidal J. Karplus W. J. Kaludjian G. (1966). “Sensitivity coefficients for the correction of quantization errors in hybrid computer systems,” in Sensitivity Methods in Control Theory. Proceedings of the International Symposium, Dubrovnik, (Pergamon Press), 197–208.

[B124] Wang T. Mo L. Mo C. Tan L. H. Cant J. S. Zhong L.. (2015). Is moral beauty different from facial beauty? Evidence from an fmri study. Soc. Cogn. Affect. Neurosci. 10, 814–823. 10.1093/scan/nsu123 25298010 PMC4448025

[B125] Weinberger N. M. (1995). Dynamic regulation of receptive fields and maps in the adult sensory cortex. Annu. Rev. Neurosci. 18, 129–158. 10.1146/annurev.ne.18.030195.001021 7605058 PMC3621971

[B126] White A. White M. (2016). Investigating practical linear temporal difference learning. arXiv [Preprint]. Available online at: https://arxiv.org/abs/1602.08771.

[B127] Widrow B. Hoff M. E. (1960). Adaptive Switching Circuits (No. TR-1553–1). Stanford Electronics Labs.

[B128] Wu C.-C. Chen C.-C. (2020). Symmetry modulates the amplitude spectrum slope effect on visual preference. Symmetry 12:1820. 10.3390/sym12111820

[B129] Xu X. Hu D. Lu X. (2007). Kernel-based least squares policy iteration for reinforcement learning. IEEE Trans. Neural Netw. 18, 973–992. 10.1109/TNN.2007.899161 17668655

[B130] Yanchang Z. Junde S. (2001). “Gdilc: a grid-based density-isoline clustering algorithm,” in Paper Presented at the 2001 International Conferences on Info-Tech and Info-Net, Beijing, China, 140–145.

[B131] Yu X. L. Lewis E. R. (1989). Studies with spike initiators: linearization by noise allows continuous signal modulation in neural networks. IEEE Trans. Biomed. Eng. 36, 36–43. 10.1109/10.16447 2784125

[B132] Zomaya A. Y. (2006). Handbook of Nature-Inspired and Innovative Computing: Integrating Classical Models with Emerging Technologies. New York, NY: Springer Science & Business Media.

